# Characterization and high-efficiency secreted expression in *Bacillus subtilis* of a thermo-alkaline β-mannanase from an alkaliphilic *Bacillus clausii* strain S10

**DOI:** 10.1186/s12934-018-0973-0

**Published:** 2018-08-11

**Authors:** Cheng Zhou, Yanfen Xue, Yanhe Ma

**Affiliations:** 1 State Key Laboratory of Microbial Resources, Institute of Microbiology, Chinese Academy of Sciences, Beijing, 100101 China; 2 National Engineering Laboratory for Industrial Enzymes, Institute of Microbiology, Chinese Academy of Sciences, Beijing, 100101 China

**Keywords:** Thermo-alkaline β-mannanase, *Bacillus clausii*, Characterization, Secreted expression, *Bacillus subtilis*

## Abstract

**Background:**

β-Mannanase catalyzes the cleavage of β-1,4-linked internal linkages of mannan backbone randomly to produce new chain ends. Alkaline and thermostable β-mannanases provide obvious advantages for many applications in biobleaching of pulp and paper, detergent industry, oil grilling operation and enzymatic production of mannooligosaccharides. However, only a few of them are commercially exploited as wild or recombinant enzymes, and none heterologous and secretory expression of alkaline β-mannanase in *Bacillus subtilis* expression system was reported. Alkaliphilic *Bacillus clausii* S10 showed high β-mannanase activity at alkaline condition. In this study, this β-mannanase was cloned, purified and characterized. The high-level secretory expression in *B. subtilis* was also studied.

**Results:**

A thermo-alkaline β-mannanase (BcManA) gene encoding a 317-amino acid protein from alkaliphilic *Bacillus clausii* strain was cloned and expressed in *Escherichia coli*. The purified mature BcManA exhibited maximum activity at pH 9.5 and 75 °C with good stability at pH 7.0–11.5 and below 80 °C. BcManA demonstrated high cleavage capability on polysaccharides containing β-1,4-mannosidic linkages, such as konjac glucomannan, locust bean gum, guar gum and sesbania gum. The highest specific activity of 2366.2 U mg^−1^ was observed on konjac glucomannan with the *K*_*m*_ and *k*_*cat*_ value of 0.62 g l^−1^ and 1238.9 s^−1^, respectively. The hydrolysis products were mainly oligosaccharides with a higher degree of polymerization than biose. BcManA also cleaved manno-oligosaccharides with polymerization degree more than 3 without transglycosylation. Furthermore, six signal peptides and two strong promoters were used for efficiently secreted expression optimization in *B. subtilis* WB600 and the highest extracellular activity of 2374 U ml^−1^ with secretory rate of 98.5% was obtained using SP_*lipA*_ and P43 after 72 h cultivation in 2 × SR medium. By medium optimization using cheap nitrogen and carbon source of peanut meal and glucose, the extracellular activity reached 6041 U ml^−1^ after 72 h cultivation with 6% inoculum size by shake flask fermentation.

**Conclusions:**

The thermo-alkaline β-mannanase BcManA showed good thermal and pH stability and high catalytic efficiency towards konjac glucomannan and locust bean gum, which distinguished from other reported β-mannanases and was a promising thermo-alkaline β-mannanase for potential industrial application. The extracellular BcManA yield of 6041 U ml^−1^, which was to date the highest reported yield by flask shake, was obtained in *B. subtilis* with constitutive expression vector. This is the first report for secretory expression of alkaline β-mannanase in *B. subtilis* protein expression system, which would significantly cut down the production cost of this enzyme. Also this research would be helpful for secretory expression of other β-mannanases in *B. subtilis*.

**Electronic supplementary material:**

The online version of this article (10.1186/s12934-018-0973-0) contains supplementary material, which is available to authorized users.

## Background

β-Mannanase, as one of the mannan-degrading enzymes, plays an important role in the hydrolysis of hemicellulose and catalyzes the cleavage of β-1,4-linked internal linkages of mannan backbone randomly to produce new chain ends [1, 2]. β-mannanases have many potential applications in industrial processes such as biobleaching of pulp and paper, detergent industry, food and feed industry, coffee processing industry, oil and gas industry, pharmaceutical applications, and second generation biofuel [1]. They are also involved in breaking down plant tissues in the cell walls [3] as well as digesting and absorbing nutrients in lower animals by degrading mannan polymers [4]. Owing to the importance and increasing application demand, many β-mannanases from different biological sources have been studied by purification, gene cloning, enzymatic characterization, structure and catalytic mechanisms, and engineering of improved features [1, 5]. Besides a few β-mannanases from plants to lower animals [3, 4], most of reported β-mannanases were from microorganisms including fungi and bacteria. Among bacteria, β-mannanases were mostly produced by Gram-positive strains like *Bacillus* species [6–14] and *Clostridia* species [15]. Only a few Gram negative strains such as *Pseudomonas* [16], *Vibrio* [17], *Thermotoga* [18], *Rhodothermus* [19], *Klebsiella* [20] and *Enterococcus* [21] have been reported to produce β-mannanase. Besides these, some β-mannanases from actinomycetes such as *Streptomyces* [22], *Cellulomonas* [23] and *Thermobifida* [24] have also been studied. For fungi, the most common mannolytic group belongs to the genus *Aspergillus* [25–29], while some species from genus *Penicillium* [30], *Trichoderma* [31] and *Neosartorya* [32] have also been investigated. Particularly, β-mannanases from extremophilic microorganisms have drawn special attention due to their unusual properties like thermo-, acid-, or alkali-tolerance which may be desirable for specific industrial processes [33]. Thermo-alkaline β-mannanases were usually used in biobleaching of pulp and paper, detergent industry, oil grilling operation and enzymatic production of mannooligosaccharides because of their high activity and good stability at high pH value and temperature [1]. Although a number of alkaline and thermostable mannanases were reported in the past decades, only a few are commercially exploited as wild or recombinant enzymes [34–36]. Therefore, there is still a pressing need to seek for new enzymes that are efficient and stable at alkaline or high temperature conditions to promote the development of the industrial application of β-mannanases.

In addition to enzyme properties, high yield is another important factor in the industrial application of enzymes. However, the yield of β-mannanases produced from native microbes, especially from extremophilic microbes, is usually low and hard to be improved, which led to the increasing attention on the heterologous production of β-mannanases in recent years [33]. Many β-mannanase genes originated from different microbes have been cloned and heterologously expressed in the frequently used protein expression systems such as *Escherichia coli* [37–39], *Pichia pastoris* [33, 40–42] and *Bacillus subtilis* [5, 43, 44] in the past decade. Compared to *E. coli*, *P. pastoris* and *B. subtilis* can secreted the enzyme directly into the culture medium, which making the downstream separation and purification much easier. However, most of the reported β-mannanases expressed in these two expression systems were acidic or neutral enzymes and only alkaline β-mannanase gene from alkaliphilic *Bacillu*s sp. N16-5 was found heterologously expressed in *P. pastoris* with the highest yield of 1114 U ml^−1^ and 6336 U ml^−1^ by shake flask and fed-batch fermentation, respectively [33, 45]. No heterologous and secreted expression of alkaline β-mannanase in *B. subtilis* was reported in the literature. In addition, *B. subtilis* possesses its own advantages in generally producing proteins with higher secretion concentration and well-characterized secretion pathways compared to *P. pastoris* [5]. Even when comparable secretion capacity occurs, *B. subtilis* still shows higher growth rate than *P. pastoris*, which makes it a priority in industrial application. On the other hand, bacteria producing β-mannanase, especially alkaline β-mannanases, are mostly confined to Gram-positives including various *Bacillus* species. Therefore, *B. subtilis* would be preferred expression system for high-level secreted production of β-mannanases including alkaline β-mannanases originated from *Bacillus* strains.

In previous research, we isolated an alkaliphilic strain identified as *Bacillus clausii* strain S10 from soda lake in Inner Mongolia, China, which showed high alkaline β-mannanase activity. In this study, we described the gene cloning, heterologous expression, purification and characterization of this alkaline β-mannanase BcManA. Further a recombinant *B. subtilis* WB600 strain containing pMA5 vector carrying the BcManA gene was constructed. Different signal peptides and strong promoters were also used for the evaluation of high-level secreted production, along with the medium and inoculum amount optimization. Finally, the highest yield of β-mannanase reported to date by shake flask was obtained.

## Methods

### Strains, plasmids, and materials

Alkaliphilic strain *B. clausii* S10 which was used to clone the mannanase gene was isolated from a soda lake in Inner Mongolia of China. *B. clausii* S10 was preserved in the China General Microbiological Culture Collection Center (CGMCC 1.15293). The plasmids and bacteria strains used in this study for gene cloning and expression are listed in Table 1. Manno-oligosaccharides (mannobiose, mannotriose, mannotetraose, mannopentose, and mannohexose) were purchased from Megazyme (Wicklow, Ireland). All the enzymes for DNA manipulations were purchased from NEB Inc. (Ipswich, MA, USA). Isopropyl-β-d-thiogalactopyranoside (IPTG), ampicillin, kanamycin and imidazole were from Amresco Inc. (Solon, OH, USA). All other chemicals were of reagent grade.

**Table 1:** Plasmids and strains used in this study

Plasmids or strains	Features	Sources
pUC118	Treated by *Bam*HI and calf intestinal alkaline phosphatase, Amp^R^	TaKaRa Co., Ltd, China
pET28a	Protein expression vector in *E. coli*, Kan^R^	Merck Co., Germany
pET28a-Man	pET28a derivative with BcManA gene without signal peptide	This work
pUC57-Simple-PlapS	pUC57 derivative with promoter P_*lapS*_ sequence	Laboratory preservation
pHT43	*E. coli*-*Bacillus* shuttle vector for protein expression in *B. subtilis*, Cm^R^	BGSC, USA
pMA5	*E. coli*-*Bacillus* shuttle vector for protein expression in *B. subtilis*, Kan^R^	BGSC, USA
pMA5-Man	pMA5 derivative with BcManA gene without signal peptide	This work
pMA5-Man1	pMA5-Man derivative, original signal peptide, SP_*ori*_	This work
pMA5-Man2	pMA5-Man derivative, SP_*lipA*_	This work
pMA5-Man3	pMA5-Man derivative, SP_*amyE*_	This work
pMA5-Man4	pMA5-Man derivative, SP_*lipB*_	This work
pMA5-Man5	pMA5-Man derivative, SP_*aprE*_	This work
pMA5-Man6	pMA5-Man derivative, SP_*amyL*_	This work
pMA5-Man2-1	pMA5-Man2 derivative, P43	This work
pMA5-Man2-2	pMA5-Man2 derivative, P_*lapS*_	This work
*B. clausii* S10	DNA source for β-mannanase gene cloning	Laboratory preservation
*E. coli* DH5α	Host for gene cloning	Transgen Biotech Co., Ltd, China
*E. coli* BL21(DE3)	Host for protein expression	Transgen Biotech Co., Ltd, China
*B. subtilis* WB600	Host for protein secreted expression, Cm^R^	Laboratory preservation
*B. subtilis* WB600-1	WB600 derivative, pMA5-Man, Kan^R^, Cm^R^	This work
*B. subtilis* WB600-2	WB600 derivative, pMA5-Man1, Kan^R^, Cm^R^	This work
*B. subtilis* WB600-3	WB600 derivative, pMA5-Man2, Kan^R^, Cm^R^	This work
*B. subtilis* WB600-4	WB600 derivative, pMA5-Man3, Kan^R^, Cm^R^	This work
*B. subtilis* WB600-5	WB600 derivative, pMA5-Man4, Kan^R^, Cm^R^	This work
*B. subtilis* WB600-6	WB600 derivative, pMA5-Man5, Kan^R^, Cm^R^	This work
*B. subtilis* WB600-7	WB600 derivative, pMA5-Man6, Kan^R^, Cm^R^	This work
*B. subtilis* WB600-8	WB600 derivative, pMA5-Man2-1, Kan^R^, Cm^R^	This work
*B. subtilis* WB600-9	WB600 derivative, pMA5-Man2-2, Kan^R^, Cm^R^	This work

### Gene cloning of BcManA

The genomic DNA of *B. clausii* S10 was extracted using Bacterial DNA Kit (OMEGA Bio-tek, Norcross, USA), and the restriction enzyme *Sau*3A was employed to obtain randomly digested chromosomal fragments. The 4.0- to 8.0-kb fragments were recovered and purified by a Gel Extraction Kit (OMEGA Bio-tek) and ligated into pUC118 vector previously digested with *Bam*HI and treated with calf intestinal alkaline phosphatase. The ligation product was then transformed into *E. coli* DH5α cells by electroporation transformation and plated onto Luria–Bertani (LB) agar plates containing 60.0 μg ml^−1^ of ampicillin and incubated at 37 °C overnight. Grown colonies were picked with sterilized toothpicks, plated in 150.0 μl LB medium containing ampicillin (60.0 μg ml^−1^) in sterilized 96-well microplates, and incubated overnight at 37 °C with shaking at 900 rpm. Fifty microliters of sterilized 60.0% glycerol was then added to each well, and plates were stored at − 80 °C to create the genomic DNA library.

Colonies in the microplate were spotted onto LB-ampicillin (60.0 μg ml^−1^) agar medium (pH 8.0) containing 0.5% locust bean gum by a sterile 96-pin replicator. After cultivating for 20 h at 37 °C, 1.0% sterile Congo red solution was softly poured onto the LB agar medium over the colonies. The colony which has transparent zone after 15 min soakage was considered to be the positive colony with mannanase activity. The inserted fragment in the positive transformant was sequenced by SinoGenoMax Co., Ltd. (Beijing, China). The gene sequence was analyzed by the NCBI BLAST program, and the SignalP 4.1 server (http://www.cbs.dtu.dk/services/SignalP/) was used for signal peptide prediction. The amino acid sequence was aligned with ClustalX.

### Expression and purification of the recombinant mature BcManA in *E. coli*

The mature BcManA-encoding gene without a signal peptide sequence was obtained by PCR using the primer pair of F (5′-CTAGCTAGCCAAAGCGGCTTTCACGTAAAAG-3′, where the underline indicates the *Nhe*I site) and R (5′-CCCAAGCTTTTAATCACGTTTGAGCCCATTTTC-3′, where the underline indicates the *Hin*dIII site). The following PCR program was used: 1 cycle of 94 °C for 3 min; 30 cycles of 94 °C for 30 s, 56 °C for 35 s, and 72 °C for 1 min; followed by a final extension at 72 °C for 10 min. The PCR product was purified using a Gel Extraction Kit (OMEGA Bio-tek) and then digested with *Nhe*I and *Hin*dIII for ligation to the pET28a which were cleaved with the same enzymes. The resultant recombinant plasmid pET28a-*manA* was transformed into *E. coli* BL21 (DE3) for gene expression. For gene expression in flask, about 2.0 ml of seed culture of recombinant *E. coli* BL21 (DE3) harboring pET28a-*manA* was inoculated into 150.0 ml of LB medium containing 60.0 μg ml^−1^ of kanamycin for cultivation. IPTG with a final concentration of 0.8 mM was added when the OD_600_ reached 0.6. And then the protein expression was induced by addition of IPTG at 37 °C for 5 h with shaking at 200 rpm.

The mature BcManA encoding gene was expressed as a recombinant fusion protein containing an N-terminal His-tag in *E. coli* and was purified to near homogeneity by a two-step process including heat treatment and nickel-nitrilotriacetic acid (Ni–NTA) affinity chromatography. Cells of about 150.0 ml culture of *E. coli* BL21 (DE3) harboring pET28a-ManA were harvested by centrifugation at 8000×*g* at 4 °C for 15 min, washed with binding buffer (20 mM Tris–HCl buffer containing 500 mM NaCl and 5 mM imidazole, pH 7.9) and then suspended in 20.0 ml of the same buffer. The suspended cells were disrupted by sonication and the supernatant was obtained by centrifugation at 16,000×*g* for 10 min at 4 °C. The supernatant was incubated at 60 °C for 20 min and then centrifuged at 16,000×*g* for 15 min at 4 °C. The second supernatant was loaded onto a pre-equilibrated Ni–NTA column filled with Ni–NTA His·Bind Resin (Novagen, CA, USA) followed by washing with 10.0 ml of binding buffer and subsequently with 15.0 ml of washing buffer (20 mM Tris–HCl buffer containing 500 mM NaCl and 60 mM imidazole, pH 7.9). Finally, the protein was eluted with 3.0 ml of elution buffer (20 mM Tris–HCl buffer containing 500 mM NaCl and 1 M imidazole, pH 7.9).

The obtained protein solution was desalted using a desalting column (GE Healthcare Bio-Sciences AB, Uppsala, Sweden) with 20 mM Tris–HCl buffer (pH 7.5). The purity of the proteins was assessed by sodium dodecyl sulfate polyacrylamide gel electrophoresis (SDS-PAGE). The protein concentration was determined by Quick Start Bradford protein assay (Bio-Rad, Hercules, CA, USA) with bovine serum albumin (0.125–1.0 mg ml^−1^) as standard.

### Activity assay of BcManA

The enzymatic activity of BcManA was determined by measuring the release of reducing sugars by the 3,5-dinitrosalicylic acid (DNS) method with mannose as the standard. The reaction mixture comprised 190.0 μl of 50 mM glycine–NaOH (pH 9.5) containing 0.5% substrate (w/v), and 10.0 μl of appropriately diluted enzyme solution. The reaction was incubated at 70 °C for 10 min and stopped by adding 200.0 μl DNS reagent, followed by heating in a boiling water bath for 5 min. The absorbance at 540 nm was measured with a Spectra Max 190 Microplate Reader (Molecular Devices, Sunnyvale, CA, USA). One unit of enzyme activity is defined as the amount of enzyme required to release 1 μmol of mannose-reducing sugar equivalents per min. The activity measurements were repeated three times.

### Effect of pH, temperature and reagents on enzyme activity and stability

The optimal pH was assayed at 70 °C in 50 mM Na_2_HPO_4_-Citric acid buffer (pH 5.5–7.5), 50 mM Tris–HCl buffer (pH 7.5–8.5), 50 mM glycine–NaOH buffer (pH 8.5–10.5) and 25 mM Na_2_CO_3_–NaOH buffer (pH 10.5–11.5) containing 0.5% konjac glucomannan (w/v). The optimal temperature was assayed at 40–90 °C for 10 min with standard reaction buffer (50 mM glycine–NaOH buffer containing 0.5% konjac glucomannan (w/v), pH 9.5). The effect of pH on enzyme stability was analyzed with enzyme being incubated in Na_2_HPO_4_–Citric acid buffer (pH 4.5–7.5), 50 mM Tris–HCl buffer (pH 7.5–8.5), 50 mM glycine–NaOH buffer (pH 8.5–10.5) and Na_2_HPO_4_–NaOH buffer (pH 11.0–12.0) at 30 °C for 6 h. Thermal stability was determined by measuring the residual activity after incubating enzyme in 50 mM glycine–NaOH buffer (pH 9.0) for different time at temperature of 50 °C, 60 °C, 70 °C and 80 °C, respectively. For determining the thermostability at 70 °C and 80 °C, the residual activity was assayed after incubation for 0.5, 1.0, 1.5, 2.0, 2.5, 3.0 and 4.0 h. For assessing thermostability at 50 °C and 60 °C, the incubation time was extended to 9.0 h. All the measurements were repeated three times.

Influence of metal ions and some reagents on enzyme activity was determined by incubating the enzyme in 1 mM of metal ions (Hg^2+^, Mg^2+^, Na^+^, Mn^2+^, Zn^2+^, Cu^2+^, Pb^2+^, Ca^2+^, Ag^+^, Fe^3+^, K^+^, Rb^+^, Cs^+^, Fe^2+^, Ni^2+^ and Co^2+^) and EDTA by measuring the relative activity under the standard assay condition. The effect of some protein denatures such as SDS, on enzyme stability was determined by the residual activity after incubation them of different concentration with the enzyme of 0.1 mg ml^−1^ at 30 °C for 30 min.

### Substrate specificity and kinetic parameters

The substrate specificity of purified recombinant BcManA was assayed by incubating the enzyme solution with 0.5% (w/v) substrates, including konjac glucomannan, locust bean gum, guar gum, sesbania gum, carboxymethyl cellulose, xylan, glucan, pectin, chitin, soluble starch and α-1,6-linked yeast mannan under standard reaction conditions (pH 9.5 and 75 °C). To detect the exoglycosidase activity, the *p*-nitrophenyl glycoside substrate (5 mM) was incubated with 1 μM enzyme for 30 min. The release of *p*-nitrophenol was monitored by measuring the absorbance at 405 nm. The hydrolysis kinetic parameters were determined at 75 °C in 50 mM glycine–NaOH buffer (pH 9.5) after a reaction time of 10 min using 0.1 μM enzyme and substrate of konjac glucomannan (0.3–5.0 mg ml^−1^), locust bean gum (0.5–6.0 mg ml^−1^), guar gum (1.0–8.0 mg ml^−1^) and sesbania gum (1.0–8.0 mg ml^−1^). The *k*_*cat*_ and *K*_*m*_ values were calculated by GraphPad Prism 5.0 software (http://www.graphpad.com/prism/) using non-linear regression. All data are expressed as the means of triplicate measurements.

### Catalytic products analysis by thin-layer chromatography (TLC)

The time course of hydrolysis was performed using 10 µM enzyme and 2% (w/v) substrate at 65 °C. For konjac glucomannan and locust bean gum, 10 µl aliquots were taken out at 0 and 90 min and boiled for 2 min to stop enzymatic hydrolysis. Whereas for manno-oligosaccharides, aliquots were taken out at 0, 10, 30, 60 and 90 min and stop the enzyme activity by boiling. The catalytic products were then analyzed by thin-layer chromatography (TLC) using Silica gel 60 F_254_ (Merck, Darmstadt, Germany). The expansion solvent used was n-butanol/H_2_O/acetic acid (2:1:1, by vol). Oligosaccharides were detected by spraying the dried plates with a solution of acetone/diphenylamine/phenylamine/phosphoric acid (100 ml:2 g:2 ml:10 ml) and heating at 110 °C for 5 min.

### Construction of plasmids for secreted expression in *B. subtilis*

For constructing the control plasmid pMA5-*manA*, the mature BcManA-encoding gene without original signal peptide was obtained by PCR using the primer pair of F1 (5′-CGCGGATCCCAAAGCGGCTTTCACGTAAAAG-3′, where the underline indicates the *Bam*HI site) and R1 (5′-CTAGCTAGCTTAATCACGTTTGAGCCCATTTTC-3′, where the underline indicates the *Nhe*I site). The PCR program was the same as the construction of plasmid pET28a-*manA*. The PCR product was purified using a Gel Extraction Kit (OMEGA Bio-tek) and then digested with *Bam*HI and *Nhe*I for ligation to the pMA5 which were cleaved with the same enzymes. Then the ligation product was transformed into *E. coli* DH5α cells and plated onto LB agar plates containing 50.0 μg ml^−1^ of ampicillin and incubated at 37 °C overnight. Besides the original peptide of BcManA, signal peptides including SP_*amyL*_, SP_*lipA*_, SP_*nprB*_, SP_*nprE*_, SP_*lipB*_ and SP_*amyE*_ from *B. subtilis* 168 were also used for secreted expression. On the other hand, other two constitutive promoters including P43 and P_*lapS*_, and the inducible promoter of P_*grac*_ were also used for BcManA expression. The fragment lengths of P43 and P_*lapS*_ were 326 bp and 308 bp, respectively. P_*grac*_ containing the promoter, *lac*O sequences, and the regulator protein *lac*I was 1548 bp and originated from the plasmid pHT43 plasmid.

The expression plasmids were constructed by PCR using the modified Gibson assembly method [46] base on the plasmid pMA5-*manA*. Primers used to amplify the signal peptide and promoter fragments for expression plasmid construction were listed in Additional file 1: Tables S1, S2. The I-5™ 2 × High-Fidelity Master Mix (Tsingke Biotech Co., Ltd, China) was used for PCR amplification. The primers for construction of plasmids with different signal peptides directly included the signal peptide sequences and the PCR protocols were as follows: denaturation at 98 °C for 2 min, followed by 30 cycles of denaturation at 98 °C for 20 s, annealing at 60 °C for 20 s and extension at 72 °C for 2 min, and a final extension at 72 °C for 5 min. The plasmid pMA5-*manA* was used as the template. The PCR product was purified by a Cycle-Pure Kit (OMEGA Bio-tek) and then digested by *Dpn*I for 6 h. Then 2 µl of this the product and 0.5 µl of Taq DNA ligase were added into 7.5 µl of assembly master mixture [46], and this mixture was incubated at 50 °C for 1 h. Then the product was directly transformed into competent *E. coli* DH5α, and positive colony samples were selected to be further validated by sequencing (Tsingke Biotech Co., Ltd, China).

For the construction of plasmids with different promoters, the PCR amplification program was divided into two parts: the amplification of promoter sequence and the pMA5-*manA* sequence. The primer pairs of F1 and R1, F2 and R2 in Additional file 1: Table S2 were used for the promoter and pMA5-*manA* sequences amplification, respectively. The PCR protocol for promoter P43 and P_*lapS*_ was as follows: denaturation at 98 °C for 2 min, followed by 30 cycles of denaturation at 98 °C for 20 s, annealing at 56 °C for 20 s and extension at 72 °C for 10 s, and a final extension at 72 °C for 5 min. For the second part of amplification, the PCR protocol was the same as that for construction of plasmids with different signal peptides. All the PCR products were purified by a Cycle-Pure Kit (OMEGA Bio-tek) and then digested by *Dpn*I for 6 h. Then each 1 µl of these corresponding products and 0.5 µl of Taq DNA ligase were added into 7.5 µl of assembly master mixture followed incubation at 50 °C for 1 h. Similarly, these reaction products were directly transformed into *E. coli* DH5α for further validation by sequencing. All the confirmed recombinant pMA5-*manA* derivative plasmids were than transformed into *B. subtilis* WB600 cells by electroporation transformation as previously [47] for secreted expression in *B. subtilis*.

### High-level secreted expression of BcManA in *B. subtilis* by shake flask

For gene expression in flask, 0.5 ml of seed culture of *B. subtilis* strains harboring appropriate expression plasmids were inoculated in 50 ml of 2 × SR medium [5] containing 60.0 μg ml^−1^ of kanamycin and 10 μg ml^−1^ of chloramphenicol in 500 ml flasks. The expression cultivation was performed at 37 °C with 230 rpm. For the determination of cell growth and β-mannanase production curves, the Horikoshi-I medium (polypeptone 5.0 g l^−1^, yeast extract 5.0 g l^−1^, glucose 10.0 g l^−1^, K_2_HO_4_ 1.0 g l^−1^, MgSO_4_·7H_2_O 0.2 g l^−1^, 10% Na_2_CO_3_ solution 100 ml l^−1^) and modified Horikoshi-I medium (glucose was replaced by 10.0 g l^−1^ of locust bean gum) were used for *B. clausii* S10, while 2 × SR medium was used for *B. subtilis* WB600 and the recombinant *B. subtilis* WB600 containing BcManA gene. The OD_600_ value was used to indicate cell biomass. The β-mannanase activity in supernatant and whole cell lysate was assayed under standard condition after cultivation. Furthermore, some cheaper nitrogen source such as soybean powder, peanut meal, peptone, NH_4_Cl and carbon source such as glucose, glycerol, soluble starch, konjac flour, corn starch and dextrin were also used to replace tryptone or yeast extract in 2 × SR medium, respectively. Similarly, 50 ml of different medium in 500 ml flasks was used for the medium component optimization and enzyme yield determination.

## Results and discussion

### Gene cloning and sequence analysis of BcManA

**Fig. 1:**
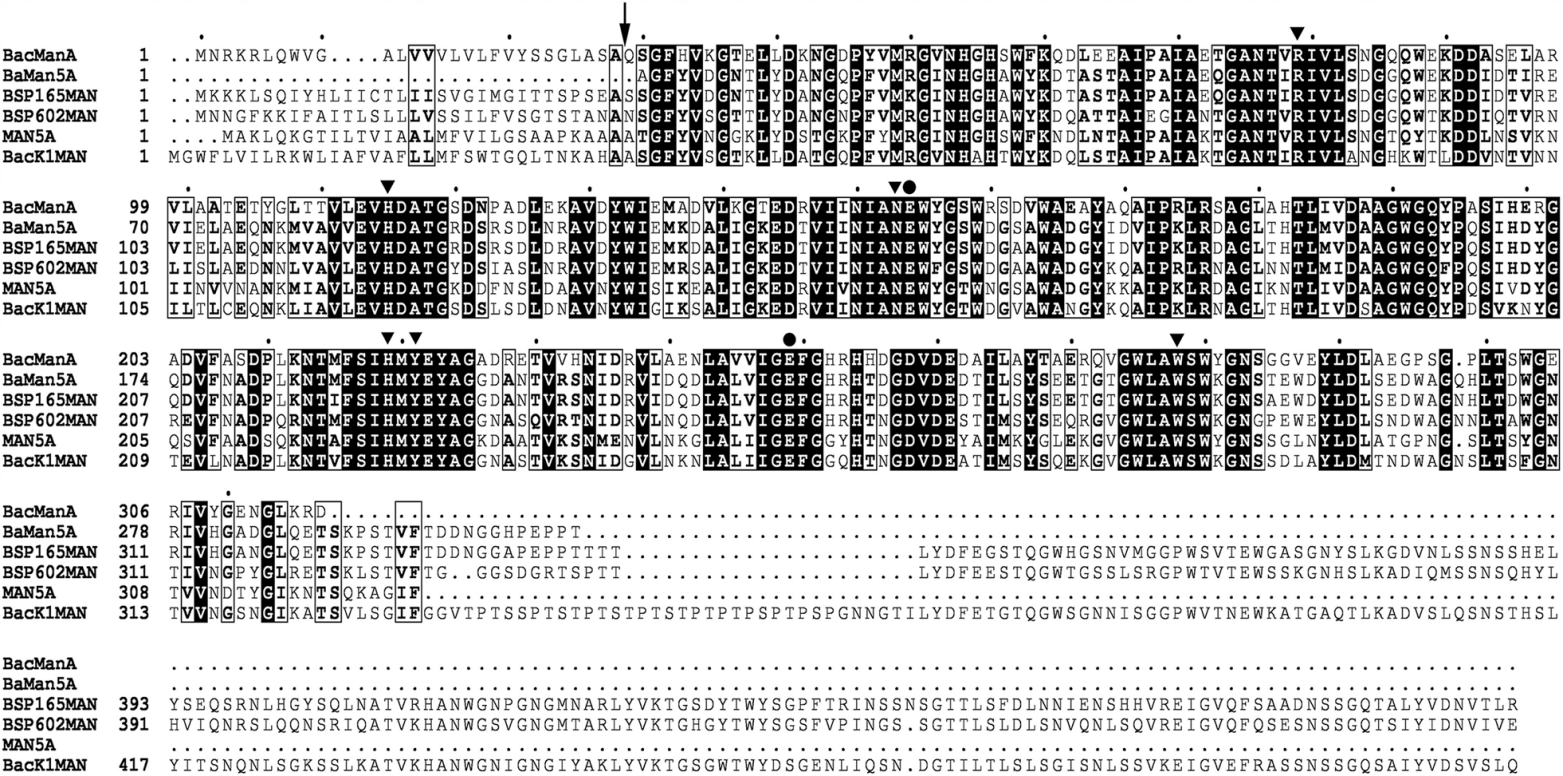
Multiple amino acid sequence alignment. The enzymes used were BcManA, BacEndoG from *Bacillus clausii* (GenBank No.WP_041823500.1), BacMan-K16 from *B. clausii* KSM-K16 (GenBank No. BAD62862.1), BaaMan5A from *B*. *agaradhaerens* (UniProtKB/Swiss-Port G1K3N4.1), BspMan-165 from *Bacillus* sp. N16-5 (GenBank No. AAT06599.1), BspMan-602 from *Bacillus* sp. JAMB-602 (BAD99527.1), BacMan-1554 from *B. circulans* CGMCC1554 (GenBank No. AAX87003.1), and BacMan-K1 from *B. circulans* K-1 (GenBank No. BAA25878.1). Strictly conserved residues are shaded black and conservatively substituted residues are boxed. Circles indicate the conserved catalytic sites. The figure was produced using ESPript 3.0 (http://espript.ibcp.fr/ESPript/ESPript/index.php). The arrow shows the cleavage site of the signal peptide of BcManA

The alkaliphilic strain *B. clausii* S10 isolated from a soda lake in Inner Mongolia of China showed high β-mannanase activity under alkaline condition. After screening approximately 4000 clones of the genomic DNA library of *B. clausii* S10, one clone with obvious transparent zone showing positive β-mannanase activity was obtained (Additional file 2: Fig. S1). Sequence analysis of the plasmid from the positive clone showed an integrated open reading frame (ORF) of 954 bp termed gene *manA* which encodes a 317-amino-acid protein named β-mannanase BcManA. The deduced protein sequence of BcManA showed the highest identity of 93% to the directly submitted endoglucanase sequence from *B. clausii* (GenBank no. WP_041823522.1) and mannan endo-1,4-β-mannosidase sequence from *B. clausii* KSM-K16 (GenBank no. BAD62862.1). However, both of these enzymes had not been reported in literature. Meanwhile, BcManA showed high homology to the characterized glycoside hydrolase family 5 mannanases from *B. agaradhaerens* (UniProtKB/Swiss-Prot G1K3N4.1), *Bacillus* sp. N16-5 (GenBank no. AAT06599.1), *Bacillus* sp. JAMB-602 (GenBank no. BAD99527.1), *B. circulans* CGMCC1554 (GenBank no. AAX87003.1), and *B. circulans* K-1 (GenBank no. BAA25878.1) with 63, 60, 60, 59, and 58% identity, respectively (Fig. 1).

In the CAZy database, the members of glycoside hydrolase family 5 have been classified in 56 subfamilies due to the divergent amino acid sequence up to now (http://www.cazy.org/GH5.html). Based on the sequence comparisons, BcManA belongs to the glycosyl hydrolase family 5, subfamily 8 (GH5_8). As previous study, eight conserved residues of all family 5 enzymes were demonstrated to be responsible for the catalytic activity [10]. The multiple sequence alignment showed that these essential active residues were also conserved in BcManA as R79, H115, N153, D154, H219, Y221, D249 and W278 (Fig. 1). Among these residues, the aspartate residues D154 and D249 are suggested to be the proton donor and the nucleophile, which was consistent with D158 and D254 in BSP165MAN belonging to GH5_8, respectively [48]. Frequently, β-mannanases carry extra non-catalytic modules which generally are the carbohydrate binding modules (CBMs) besides the catalytic module [1]. The CBM are thought to enhance enzyme activity toward cellulose conjugated mannan. However, no obvious CBM modules were found in BcManA or the five high homologous mannanases by the sequence analysis. Moreover, as an extracellular enzyme, the BcManA protein also includes a typical amino-terminal signal sequence with 28 amino acids (Fig. 1), which revealed that the BcManA mature protein contained 289 amino acids and had a calculated molecular mass of 34.3 kDa and an isoelectric point of 4.6.

### Expression in *E. coli* and purification of recombinant BcManA

**Fig. 2:**
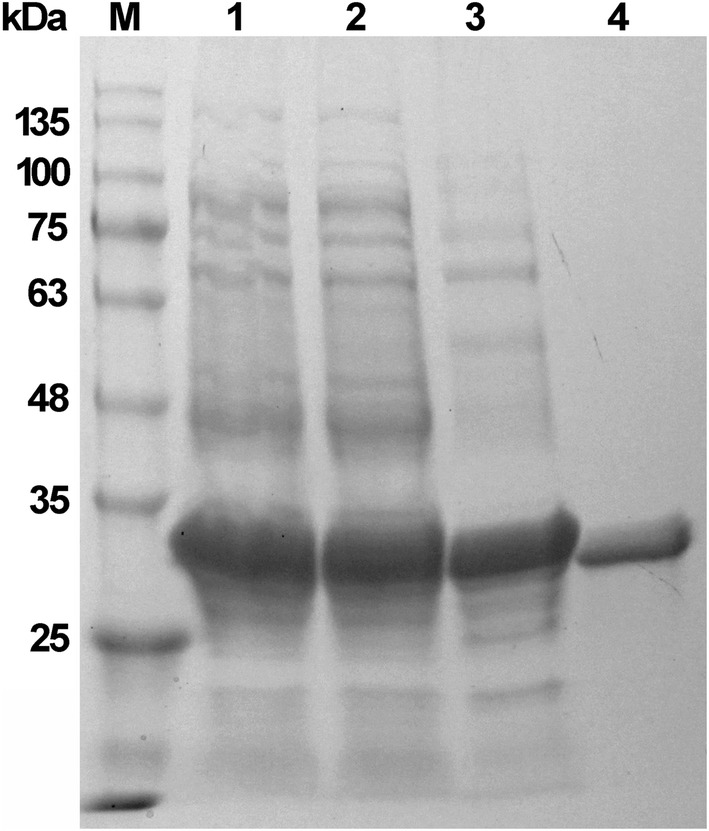
SDS-PAGE analysis of purified recombinant BcManA. Lane 1, crude extract from *E. coli* BL21(DE3) harbouring pET28a-*manA*; lane 2, supernatant of the crude extract from *E. coli* BL21(DE3) harbouring pET28a-*manA*; lane 3, supernatant after heating treatment from *E. coli* BL21(DE3) with pET28a-*manA*; lane 4, purified enzyme by His-Tag affinity chromatography; lane M, molecular weight marker

Using the shake-flask cultivation in LB medium, the total activity of the mature BcManA reached 343 U ml^−1^ with extracellular activity of 5 U ml^−1^ after 5 h induction using locus bean gum as substrate. The recombinant enzyme was purified by the above two-step process with a recovery activity of 40.9% and a final purity of about 97.5%. SDS-PAGE analysis showed that the purified recombinant mature BcManA has a molecular mass about 34 kDa (Fig. 2) which was consistent with the calculated molecular weight of 34.3 kDa. The reported *Bacillus* β-mannanases showed the majority of molecular mass from 30 to 70 kDa (Table 2). The smallest and largest β-mannanases reported to date were found to be 22 kDa and 130 kDa from *B. halodurans* PPKS-2 and *Bacillus* sp. JAMB-750, respectively [49, 50]. Moreover, after heating at 60 °C for 20 min, almost no BcManA activity loss was found, while most of the *E. coli* proteins were denatured, which indicated that an appropriate heat treatment is efficient for the purification process. The specific activity of the purified BcManA was 2087.1 and 2366.2 U mg^−1^ with locust bean gum and konjac glucomannan as substrate, respectively. This activity was lower than the homologous β-mannanases of BSP165MAN from *Bacillus* sp. N16-5 (5065.0 U mg^−1^) [10], MAN5A from *B. circulans* CGMCC1554 (4839.0 U mg ^−1^) [51] and BacK1MAN from *B. circulans* K-1 (3140.0 U mg ^−1^) [52], but higher than BSP602MAN from *Bacillus* sp. strain JAMB-602 (287.0 U mg ^−1^) [9]. In addition, although there are some β-mannanases which specific activity was higher than BcManA, the specific activity of BcManA was still higher than most other reported alkaline and *Bacillus* β-mannanases (Table 2).

**Table 2:** Physicochemical properties of characterized *Bacillus* β-mannanases

Bacteria strain	Mol. wt (kDa)	Optimum		Stability		Substrate preferred	Specific activity (U mg^−1^)	References
		pH	Temp	pH	Temp			
*B. clausii* S10	34	9.5	75	7.0–11.5	≤ 70	KGM	2366.2	This work
*B. subtilis* CSB31	47	12.5	60	5.8–12.5	≤ 65	LBG	1796.1	[53]
*B. halodurans* PPKS-2	22	11.0	70	8.0–12.0	≤ 70	CG	776.0	[49]
*Bacillus* sp. JAMB-750	130	10.0	55	6.0–10.5	≤ 50	LBG	36.3	[50]
*B. cereus* N1	63	10.0	50	NA	NA	NA	628.3	[54]
*Bacillus* sp. N16-5	55	9.5	70	8.5–10.0	≤ 60	LBG	5065.0	[10]
*Bacillus* sp. JAMB-602	50	9.0	65	6.0–11.0	≤ 50	LBG	287.0	[9]
*Bacillus* sp. AM-001	58	9.0	60	8.0–9.0	≤ 50	NA	NA	[74]
*B. nealsonii* PN-11	50	8.8	65	5.0–10.0	≤ 70	LBG	2288.9	[13]
*B. circulans* CGMCC1554	32	7.6	60	6.0–10.0	≤ 50	LBG	4839.0	[51]
*B. circulans* CGMCC1416	31	7.6	58	7.0–9.0	≤ 50	LBG	481.6	[12]
*Bacillus* sp. CSB39	30	7.5	70	4.6–11.0	≤ 70	LBG	1063.9	[68]
*Bacillus* sp. W-2	36	7.0	70	5.0–10.0	≤ 60	LBG	710.0	[7]
*B. subtilis* BCC41051	38	7.0	60	5.0–11.5	≤ 60	NA	3169.0	[76]
*B. licheniformis* DSM13	45	7.0	50	6.0–9.0	≤ 55	KGM	1672.0	[37]
*B. licheniformis*	NA	7.0	60	5.0–9.0	≤ 60	LBG	4341.0	[8]
*B. subtilis* H5	37	7.0	55	6.0–7.5	≤ 45	NA	1900.0	[72]
*B. circulans* M-21	33	7.0	50	6.0–9.0	≤ 40	KGM	19,373.3^a^	[65]
*B. circulans* K-1	62	6.9	65	NA	NA	KGM	3100.0	[52]
*B. stearothermophilus*	76	6.5	70	NA	≤ 70	LBG	100.0	[6]
*B. pumilus* GBSW19	44	6.5	65	5.0–11.0	≤ 60	LBG	1021.0	[14]
*Bacillus sp.* HJ14	40	6.5	65	5.0–10.0	≤ 60	LBG	443.4	[70]
*Bacillus* sp. MG-33	NA	6.5	65	6.5	≤ 60	LBG	591.7	[69]
*B. subtilis* G1	41	6.5	45	5.0–9.0	≤ 50	LBG	2718.0	[80]
*B. subtilis* YH12	40	6.5	55	4.5–8.0	≤ 60	LBG	7302.4	[71]
*B. subtilis* B36	38	6.4	50	5.0–8.0	≤ 60	LBG	927.8	[11]
*B. subtilis* BE-91	28	6.0	65	4.5–7.0	≤ 70	KGM	79.9	[62]
*Bacillus sp.* SWU60	38	6.0	60	5.0–9.0	≤ 60	KGM	14.0	[63]
*B. subtilis* TBS2	42	6.0	60	2.0–8.0	≤ 100	LBG	1653.0	[60]
*B. subtilis* WL-3	38	6.0	60	NA	≤ 60	LBG	5900.0	[43]
*B. subtilis* MAFIC-S11	40	6.0	50	2.0–7.0	< 60	LBG	3706.0	[42]
*B. circulans* NT 6.7	40	6.0	50	NA	NA	LBG	295.0	[38]
*B. subtilis* WY34	40	6.0	65	5.5–10.1	≤ 60	LBG	8302.4	[67]
*B. subtilis* WL-7	38	6.0	55	4.5–9.0	≤ 60	LBG	10,080.0	[73]
*Bacillus* sp. MSJ-5	41	5.5	50	5.0–9.0	≤ 65	LBG	5383.0	[55]
*B. subtilis* NM-39	38	5.0	55	4.0–9.0	≤ 55	LBG	108.0	[56]

*NA* data not available

^a^The activity was defined as the amount of enzyme that released 1 µg reducing sugar from mannan per minute

### Biochemical characterization of recombinant BcManA

**Fig. 3:**
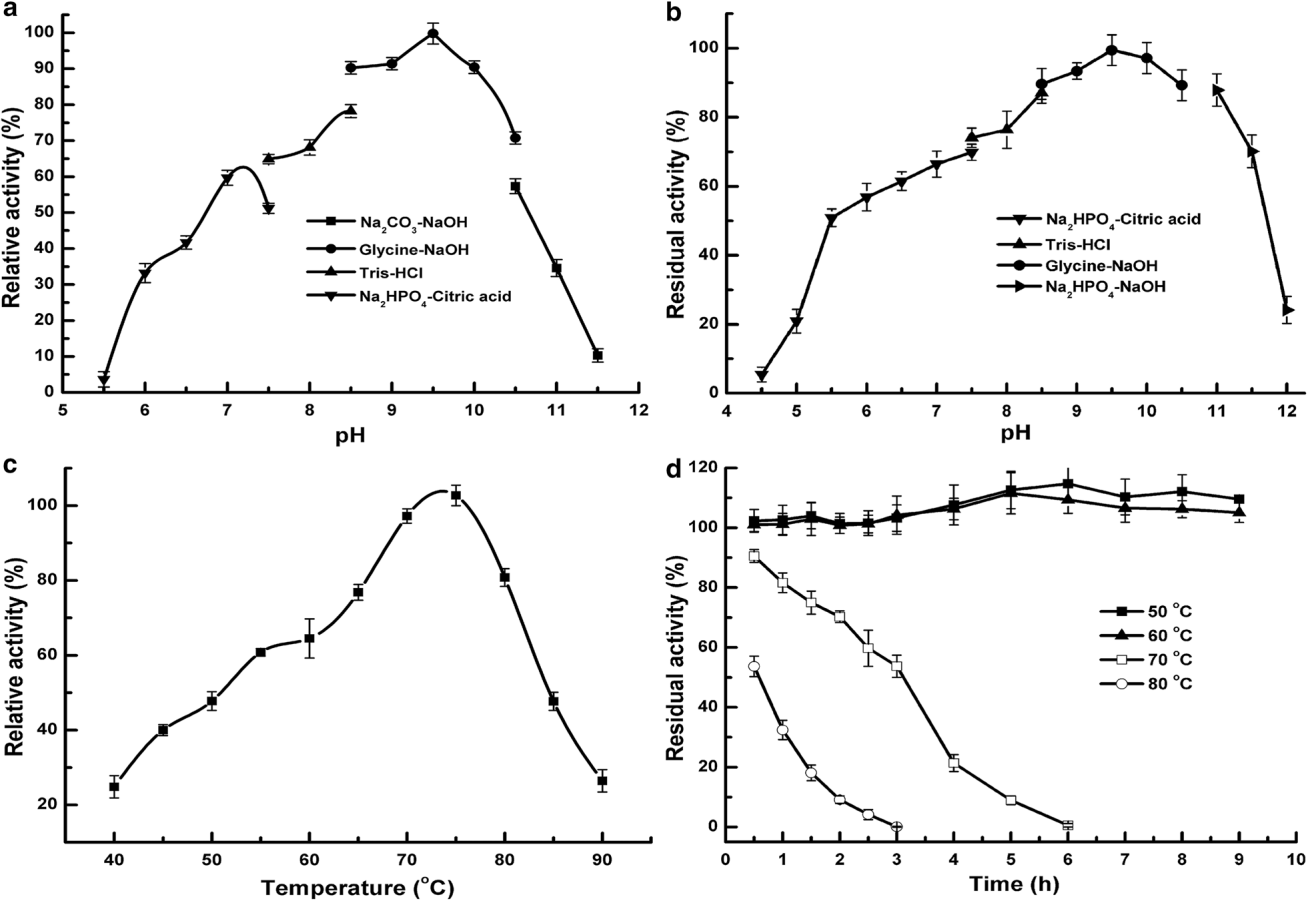
pH and temperature profiles of the purified recombinant BcManA. **a** Optimal pH: the buffer used was 50 mM Na_2_HPO_4_–Citric acid buffer (pH 5.5–7.5, black down-pointing triangle), 50 mM Tris–HCl buffer (pH 7.5–8.5, black up-pointing triangle), 50 mM glycine–NaOH buffer (pH 8.5–10.5, black circle) and 25 mM Na_2_CO_3_–NaOH buffer (pH 10.5–11.5, black square);** b** pH stability: the buffer with different pH was 50 mM Na_2_HPO_4_–Citric acid buffer (pH 4.5–7.5, black down-pointing triangle), 50 mM Tris–HCl buffer (pH 7.5–8.5, black up-pointing triangle), 50 mM glycine–NaOH buffer (pH 8.5–10.5, black circle) and Na_2_HPO_4_–NaOH buffer (pH 11.0–12.0, black right-righting pointer);** c** optimal temperature;** d** thermal stability: the enzyme was incubated at 50 °C (black square), 60 °C (black up-pointing triangle), 70 °C (white square) and 80 °C (white circle) in 50 mM glycine–NaOH buffer (pH 9.0). All of the activities were measured under standard enzyme assay conditions with locust bean gum as the substrate. Values are expressed as the means of three experiments. Error bars represent standard deviations

The effect of pH and temperature on the recombinant BcManA was studied using locust bean gum as substrate. The recombinant BcManA had a pH optimum at 9.5 which was similar to β-mannanases from *Bacillus* sp. N16-5 [10] and *Bacillus* sp. strain JAMB-602 [9] but lower than those extreme alkaline enzymes from *Streptomyces* sp. CS428 (pH 12.5) [22], *B. subtilis* subsp. *inaquosorum* CSB31 (pH 12.5) [53], *B. halodurans* PPKS-2 (pH 11.0) [49], *Bacillus* sp. JAMB-750 (pH 10.0) [50] and *B. cereus* N1 (pH 10.0) [54]. By setting the activity at pH 9.5 as 100.0%, BcManA showed relative high activity at pH 7.0–10.5 and more than 50.0% of full activity was retained at that pH range. However, the relative activity declined rapidly at pH below 6.5 or above 10.5. There was only 32.5% and 34.1% of relative activity at pH 6.0 and 11.0 (Fig. 3a). BcManA was stable over an alkaline pH range from 6.0 to 11.5 (Fig. 3b), in which more than 55.0% of the original enzyme activity was retained after 6 h at 30 °C. However, the residual activity declined certainly when the pH values were lower than 5.5 or higher than 11.5, and only 20.5% and 25.8% of original activity was retained after pretreatment at pH 5.0 and 12.0, respectively (Fig. 3b). These indicated that BcManA was a typical moderate alkaline β-mannanase. An extremely alkaline mannanase (MnB31) from *B. subtilis* subsp. *inaquosorum* CSB31 was found to have the highest optimal pH of 12.5 and to be stable at pH range from 5.8 to 12.5, which was the most alkaline β-mannanase reported to date [53]. In addition, almost all the reported *Bacillus* β-mannanases displayed optimal pH at the range from 6.0 to 12.5 (Table 2), which indicated that *Bacillus* strains were the main sources of neutral and alkaline β-mannanases. Only the β-mannanases from *Bacillus* sp. MSJ-5 [55] and *B. subtilis* NM-39 [56] showed certain acidic enzymatic characteristics. Relative to neutral and alkaline β-mannanases, acidic β-mannanases mostly come from fungus, such as those from *Penicillium oxalicum* GZ-2 [30], *Neosartorya fischeri* P1 [32], *Aspergillus niger* LW-1 [29], *Gloeophyllum trabeum* [57], *Phialophora* sp. p13 [58], and *Bispora* sp. MEY-1 [59] showing optimal pH of 4.0, 4.0, 3.5, 2.5, 1.5, 1.5, respectively.

Generally, most of reported β-mannanases showed optimal temperature of 40–75 °C [60]. There were only few β-mannanases that had optimal temperature above 75 °C, such as Man5 from *Thermotoga maritime* [18], MAN-P from *A. niger* [61], rMan5P1 from *N. fischeri* [32], Man5XZ3 from *A. nidulans* XZ3 [25], and ManBK01 from *A. niger* BK01 [27]. Man5 had optimal temperature of 90 °C which was the highest reported to date, whereas the other four fungus β-mannanases showed the highest activity at 80 °C. The optimal temperature of BcManA was 75 °C and more than 40.0% of the activity was maintained in the range of 50–85 °C (Fig. 3c). Although this optimal reaction temperature was lower than those five thermostable β-mannanases with pH optimum of 4.0–7.0, it was still the highest of all reported *Bacillus* and alkaline β-mannanases including the four highly homologous enzymes (Table 2). Previous research indicated that most β-mannanases were stable at temperatures equal or less than 60 °C and the enzyme activity decreased rapidly at higher temperatures [60]. The BcManA showed good thermostability and almost no activity loss was found after 9 h incubation at 50 °C and 60 °C (Fig. 3d). Actually, no obvious activity loss was detected even after incubating BcManA at 50 °C for 52 h (data not shown). For incubation at 60 °C, the obvious activity loss appeared after incubation for more than 36 h, and about 68.1% of the original activity retained after incubation for 48 h. There was even about 80.0% and more than 50.0% of the original activity retained after incubation at 70 °C for 1 h and 3 h, respectively. The residual activity decreased rapidly when incubation at 80 °C and only about 50.0% and 32.0% activity retained after 0.5 and 1 h incubation, respectively. Although the three fungi β-mannanases including rMan5P1, Man5XZ3 and ManBK01 had higher optimal temperature, they showed less thermostability than BcManA. Only 50.0% activity was retained after incubating rMan5P1 at 70 °C for 10 min [32]. Meanwhile, about 70.0% activity of Man5XZ3 retained after 180 min incubation at 60 °C [25], whereas only 50.0% activity of ManBK01 retained after 15 min incubation at 80 °C [27]. Only MAN-P showing 61.0% residual activity after incubation at 80 °C for 120 min [61] and Man5 with half-life of 38 min at 90 °C [18] displayed better thermostability than BcManA. Furthermore, by comparison with other β-mannanases, the thermostability of BcManA was also better than all reported alkaline β-mannanases and most of *Bacillus* β-mannanases (Table 2) except for that from *B. subtilis* TBS2 which showed about 60% activity retained at 80 °C [60]. β-mannanases have many applications in the industrial processes, such as paper industry, food industry, oil drilling, detergent industry, hydrolysis of coffee extract, textile and cellulosic fiber processing industry [1]. Among these applications, the activity and stability of β-mannanase at alkaline and thermal condition was crucial especially for enzymatic breaking of hydraulic fracturing fluids in oil drilling industry, biobleaching of pulp and paper in the paper industry, and enzymatic scouring and desizing processes in the textile industry [10, 34]. Overall consideration of pH and temperature performance, BcManA distinguished from other reported β-mannanases and was a promising thermo-alkaline β-mannanase for potential industrial application.

The effect of metal ions and chemicals on the activity of BcManA was evaluated under standard condition (Table 3). Hg^2+^, Pb^2+^, Ag^+^, Zn^2+^, Fe^2+^, and Fe^3+^ strongly inhibited the activity of BcManA, whereas Cu^2+^, Mn^2+^, Ni^2+^ and Co^2+^ partially inhibited BcManA. Heavy metal irons are known to have strong affinities for sulfhydryl (-SH) groups and normally act as an irreversible inhibitor for enzyme activity. Similarly, heavy metal irons such as Ag^2+^ and Hg^2+^ also strongly inhibited activity of other highly homologous β-mannanases except for BacK1MAN which activity was not affected by Ag^2+^ [52]. Furthermore, Pb^2+^ showed strong inhibition to BcManA and BSP602MAN but no affection on BSP165MAN [9, 10]. Also Fe^2+^ and Fe^3+^ which strongly inhibited BcManA just showed unobvious affection on BSP165MAN and BacK1MAN [10, 52]. The other tested metal ions and SDS did not markedly affect BcManA activity except for Ca^2+^ which activated the activity about 12.3%. Different from BcManA, the activity of MAN5A was strongly inhibited by SDS [51]. BcManA also showed good tolerance to EDTA and urea, with about 44.0% and 22.7% activation obtained after treatment with 1.0 mM EDTA and 5.0 M urea at 30 °C for 30 min, respectively (Table 3). Differently, only 27.0% original activity of MAN5A was retained after incubation with 1 mM EDTA [51]. However, BcManA was sensitive to guanidine hydrochloride and only 20.5% activity retained following incubation in 1.0 M guanidine hydrochloride (Table 3).

**Table 3:** Effects of metal ions and reagents on BcManA activity

Compound (1 mM)	Relative activity (%)	Compound (1 mM)	Relative activity (%)
Control	100.0	Co^2+^	32.0
Cu^2+^	75.1	Fe^3+^	0.0
Fe^2+^	1.5	Rb^+^	105.7
Hg^2+^	0.0	Cs^+^	92.1
Mg^2+^	108.4	K^+^	97.4
Mn^2+^	44.8	Na^+^	98.3
Ni^2+^	22.1	EDTA	144.0
Pb^2+^	1.0	SDS (1%)	106.2
Zn^2+^	1.1	Urea (5 M)	122.7
Ag^+^	0.0	GH (1 M)	20.5
Ca^2+^	112.3	–	–

*GH* guanidine hydrochloride

### Substrate specificity and kinetic parameters

The hydrolytic activity of the recombinant BcManA towards various substrates was evaluated at 75 °C and pH 9.5. BcManA showed substantial activity towards polysaccharides containing β-1,4-mannosidic linkages such as konjac glucomannan (KGM), locust bean gum (LBG), guar gum (GG) and sesbania gum (SG), but no activity was observed towards carboxymethyl cellulose, xylan, glucan, pectin, chitin, soluble starch, or α-1,6-linked yeast mannan. Also, BcManA showed no activity on p-nitrophenyl β- and α-mannosides, β- and α-galactosides, and β- and α-glucosides, which indicated that BcManA was unable to cleave terminal mannosides. As shown in Table 4, the highest activity of 2366.2 U mg^−1^ was obtained towards konjac glucomannan. BcManA also showed about 88.2% activity on LBG relative to KGM (taken as 100.0%), whereas the activity on SG and GG was only 26.0% and 18.1% relative to that on KGM, respectively. KGM is a glucomannan with glucose/mannose ratio of 1:1.6, whereas LBG and SG (or GG) are galactomannan with a galactose/mannose ratio of 1:4 and 1:2, respectively [1]. This indicated that the BcManA activity seems to be limited by the side group substitution of substrates and BcManA prefers to catalyze mannan substrates with lower substitution rate. Similar specificity was also found in some β-mannanases such as those from *B. circulans* K-1 [52], *B. subtilis* BE-91 [62], *B. licheniformis* DSM13 [37], *Bacillus* sp. MSJ-5 [55], *Bacillus* sp. SWU60 [63], and *Reinekea* sp. KIT-YO10 [64] except for those enzymes from *B. licheniformis* DSM13, *Bacillus* sp. SWU60 and *B. circulans* K-1 showing almost no activity on guar gum. However, most of reported *Bacillus* β-mannanases including the five enzymes showing high sequence identity with BcManA still showed the highest activity towards LBG instead of KGM (Table 2).

**Table 4:** Substrate specificity and kinetic parameters of BcManA

Substrate	Specific activity (U mg^−1^)	Relative activity (%)	Mean *K*_*m*_ (g l^−1^) ± SD	Mean *k*_*cat*_ (s^−1^) ± SD	Mean *k*_*cat*_/*K*_*m*_ (l s^−1^ g^−1^) ± SD
Konjac glucomannan	2366.2	100.0	0.62 ± 0.05	1238.9 ± 13.2	1998.2 ± 21.4
Locust bean gum	2087.1	88.2	1.68 ± 0.04	1376.1 ± 15.1	819.1 ± 25.7
Guar gum	428.3	18.1	3.16 ± 0.06	109.9 ± 2.5	34.8 ± 1.3
Sesbania gum	615.2	26.0	5.56 ± 0.11	164.7 ± 2.9	29.2 ± 1.1

The *K*_*m*_ values of BcManA for KGM, LBG, GG and SG were 0.62, 1.68, 3.16 and 5.56 g l^−1^, resulting in *k*_*cat*_/*K*_*m*_ values of 1998.2, 819.1, 34.8 and 29.2  l s^−1^ g^−1^, respectively (Table 4), which further confirming the preferred activity towards KGM over the other substrates tested. For substrate KGM, BcManA showed a *K*_*m*_ value which was similar to β-mannanase from *A. niger* BK01 (0.60 g l^−1^) [27] but lower than that of other β-mannanases including those from *Phialophora* sp. P13 (1.30 g l^−1^) [58], *Reinekea* sp. KIT-YO10 (1.60 g l^−1^) [64], *B. subtilis* BE-91 (1.75 g l^−1^) [62], *P. oxalicum* GZ-2 (2.10 g l^−1^) [30], *Bispora* sp. MEY-1 (2.63 g l^−1^) [59], *P. pinophilum* C1 (4.80 g l^−1^) [66], *Bacillus* sp. MSJ-5 (7.50 g l^−1^) [55], *B. subtilis* WY34 (10.50 g l^−1^) [67], and *B. licheniformis* DSM13 (14.9 g l^−1^) [37]. This indicated that BcManA had higher substrate affinity and maximum catalytic efficiency at low KGM concentration. Although the *K*_*m*_ value of BcManA for the standard substrate LGB was higher than that of some β-mannanases from *B. subtili*s subsp. *inaquosorum* CSB31 (0.043 g l^−1^) [53], *Bacillus* sp. CSB39 (0.082 g l^−1^) [68], *Bacillus* sp. MG-33 (0.20 g l^−1^) [69], *N. fischeri* P1 (0.83 g l^−1^) [32], *A. nidulans* XZ3 (0.90 g l^−1^) [25], *Streptomyces* sp. CS428 (1.01 g l^−1^) [22], and *A. niger* LW-1 (1.10 g l^−1^) [29], it was also lower than that of most reported β-mannanases such as those from *Bacillus* sp. HJ14 (2.20 g l^−1^) [70], *Phialophora* sp. P13 (2.50 g l^−1^) [58], *Bacillus* sp. JAMB-602 (3.10 g l^−1^) [9], *B. halodurans* PPKS-2 (3.85 g l^−1^) [49], *P. pinophilum* C1 (5.60 g l^−1^) [66], *B. subtilis* BE-91 (7.14 g l^−1^) [62], *B. nealsonii* PN‑11 (7.22 g l^−1^) [13], *B. subtilis* WY34 (7.60 g l^−1^) [67], *B. subtilis* MAFIC-S11 (8.00 g l^−1^) [42], *Bacillus* sp. MSJ-5 (11.67 g l^−1^) [55], and *B. subtilis* YH12 (30.00 g l^−1^) [71]. This indicated that BcManA also showed high LBG affinity among the reported β-mannanases. In addition, BcManA showed a high *k*_*cat*_ value of 1238.9 s^−1^ for KGM which was only lower than that of MAN5 from *Bacillus* sp. MSJ-5 (3.8 × 10^4^ s^−1^) which was an acidic and mesophilic β-mannanase. Higher *k*_*cat*_ value indicates its better hydrolytic efficiency [52]. Therefore, BcManA not only showed the highest KGM affinity but also was more catalytically efficient for KGM than other reported alkaline and thermostable β-mannanases.

### Analysis of hydrolysis product by TLC

**Fig. 4:**
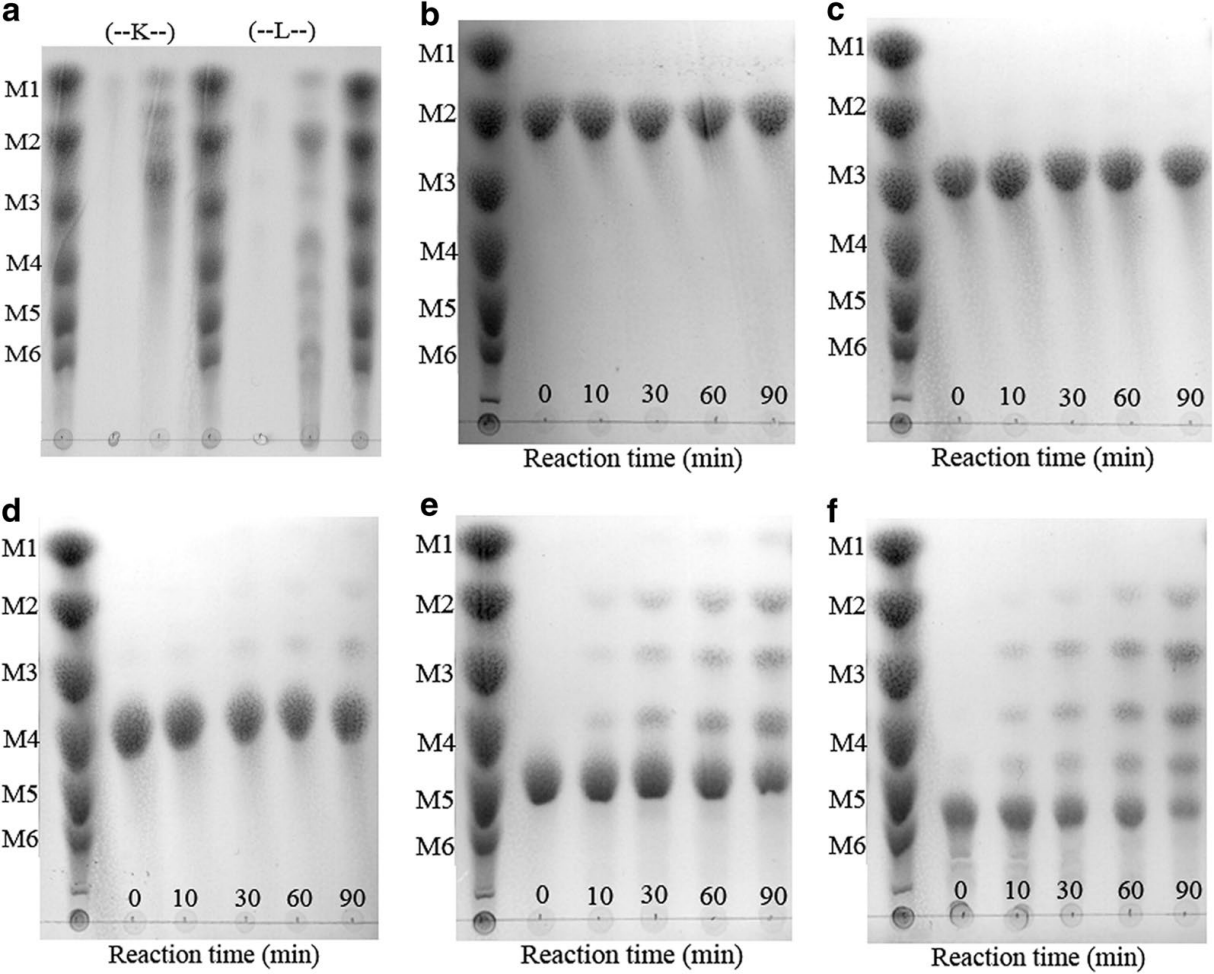
Thin-layer chromatography (TLC) analysis of hydrolysis products from mannopolysaccharides and manno-oligosaccharides. **a** Hydrolysis products from locust bean gum (L) and konjac glucomannan (K); **b**–**f** time course of manno-oligosaccharide degradation. **b** mannobiose; **c** mannotriose; **d** mannotetraose; **e** mannopentose; **f** mannohexose. M1–M6, the standard marker, mixture of manno-oligosaccharides. The numbers on the graphs indicate the reaction time

The hydrolysis products from LBG and KGM by BcManA were analyzed by TLC. A mixture of mannose oligosaccharides consisting of mannose (M1), mannobiose (M2), mannotriose (M3), mannotetraose (M4), mannopentose (M5), and mannohexose (M6) were used as standard markers. As shown in Fig. 4a, the products hydrolysed from LBG to KGM were mainly oligosaccharides with various sizes after 90 in cumulative action, which indicated that BcManA was an endo-acting β-mannanase. In addition, small amount of mannose was also found in the hydrolysed products, which was similar to the β-mannanases from *Bacillus* sp. N16-5 [10], *Bacillus* sp. W-2 [7], *B. subtilis* YH12 [71], *B. subtilis* BS5 [41], *B. subtilis* WL-3 [43], *B. subtilis* NM-39 [56], *B. circulans* CGMCC1554 [51], *Bacillus* sp. MG-33 [69] and *B. subtilis* 5H [72], but different from those from *B. subtilis* WY34 [67] and *B. subtilis* subsp. *inaquosorum* CSB31 [53] which did not produce mannose.

The catalytic activity of BcManA on the manno-oligosaccharides was also assayed. BcManA showed no activity on M2 (Fig. 4b) and very low activity on M3 (Fig. 4c). For M4–M6, the activity increased along with the degree of polymerization (DP) increase and the efficient hydrolytic activity was found on M5 and M6 (Fig. 4c–f). The products mainly were manno-oligosaccharides with DP of ≥ 2 and only very few mannose was detected. These were similar to the β-mannanases from *B. subtilis* WL-3 [43], *B. subtilis* WL-7 [73] and *Bacillus* sp. AM-001 [74], but different from those from *B. subtilis* WY34 [67], *Bacillus* sp. JAMB-602 [9], *Enterococcus casseliflavus* FL2121 [21], and *Pseudomonas* sp. strain PT-5 [16] which only hydrolysed manno-oligosaccharides with DP ≥ 4. In addition, no products with higher DP than the substrates were found, which indicated that BcManA had no transglycosylation activity. Whereas, some β-mannanases such as that from *B. subtilis* WL-3 showed transglycosylation activity when manno-oligosaccharides with DP ≥ 3 as substrates [43]. Manno-oligosaccharides are generally supplemented in animal feed to improve digestibility and maintain gastrointestinal status, and also used as food additives for humans to promote selective growth of beneficial intestinal microflora [53, 67]. As previous reports, the production of manno-oligosaccharides by acid hydrolysis of mannan is neither environmentally friendly nor cost-effective, whereas the use of alkali-thermostable endo mannanase will further improve the yield of manno-oligosaccharides due to high solubility of mannan in alkaline media and reduction in viscosity of the reaction mixture [22]. So BcManA could be a good candidate for manno-oligosaccharides production from cheap mannan such as konjac power and locust bean gum under alkaline and high temperature condition efficiently.

### Signal peptides and promoters determination for efficient secreted expression of BcManA in *B. subtilis*

**Fig. 5:**
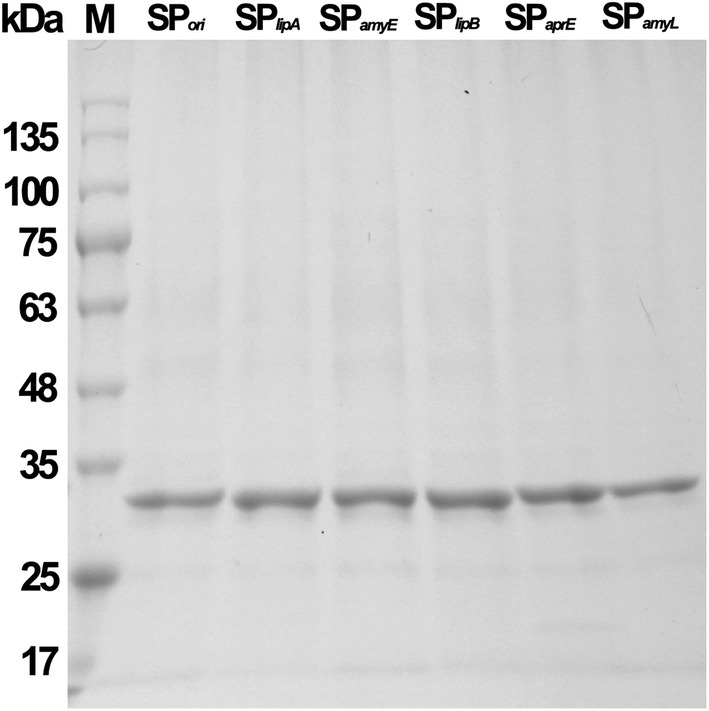
SDS-PAGE analysis of BcManA in the culture supernatant of the recombinant *B. subtilis* WB600 strains containing expression vectors with different signal peptides. Lane M, molecular weight marker

Compared to *E. coli* which was extensively used for protein expression, *B. subtilis* displayed significant advantages in efficient secretion capacity of functional extracellular proteins directly into culture medium, which made downstream separation and purification processes much more simple [5, 44]. In this study, we used the *B. subtilis* WB600, a protease-deficient strain, for secreted expression of BcManA. The multicopy plasmid pMA5 which containing the constitutive promoter P_*hpaII*_ was used as the expression vector. Firstly, a suitable and efficient signal peptide is necessary for protein secretory expression in *B. subtilis* [5]. Five different signal peptides (SP_*lipA*_, SP_*amyE*_, SP_*lipB*_, SP_*aprE*_, SP_*amyL*_) from the general secretory (Sec) pathway in *B. sutilis* and the original signal peptide of BcManA (SP_*ori*_) were selected for BcManA secretory expression. After 48 h cultivation in 2 × SR medium by flask shaking with 1% (v/v) inoculation of overnight culture, significant protein bands were found on SDS-PAGE from the culture supernatant (Fig. 5). All the protein banks showed molecular weight about 34 kDa which was in accordance with the calculated molecular weight of the mature BcManA. This indicated that all of the used signal peptides secreted BcManA into the medium successfully. Among these six signal peptides, different level of extracellular activity was detected and the highest activity of 763 U ml^−1^ was obtained with secretory rate of 95.8% by *B. subtilis* WB600-3, which indicated that SP_*lipA*_ was the most efficient signal peptide for BcManA expression in *B. subtilis* WB600 in this study (Table 5). This was similar to the β-mannanase from *B. licheniformis* DSM13 which also showed the highest extracellular activity of 533 U ml^−1^ after 72 h cultivation by SP_*lipA*_ which however, was lower than BcManA [5]. Unexpectedly, *B. subtilis* WB600-1 containing the expression vector without signal peptide also showed high extracellular activity of 571 U ml^−1^ after 48 h cultivation with the secretory rate of 59.6%. We detected the β-galactosidase activity in the culture supernatant and the whole cell lysate of *B. subtilis* WB600-1, and about 60% of total β-galactosidase activity (the activity in the whole cell lysate was set to 100%) was found in culture supernatant (data not shown). This result indicated that the high extracellular β-mannanase activity in *B. subtilis* WB600-1 culture supernatant might be due to cell lysis after 48 h cultivation.

**Table 5:** Activity of BcManA secreted by different recombinant strains

Strains	Signal peptide	Promoter	Activity (U ml^−1^)
*B. subtilis* WB600-1	No SP	P_*hpaII*_	571
*B. subtilis* WB600-2	SP_*ori*_	P_*hpaII*_	418
*B. subtilis* WB600-3	SP_*lipA*_	P_*hpaII*_	763
*B. subtilis* WB600-4	SP_*amyE*_	P_*hpaII*_	485
*B. subtilis* WB600-5	SP_*lipB*_	P_*hpaII*_	589
*B. subtilis* WB600-6	SP_*aprE*_	P_*hpaII*_	448
*B. subtilis* WB600-7	SP_*amyL*_	P_*hpaII*_	360
*B. subtilis* WB600-8	SP_*lipA*_	P43	908
*B. subtilis* WB600-9	SP_*lipA*_	P_*lapS*_	122

Besides the signal peptide, the promoter was another factor for efficient protein expression. So using strong promoters was one of the most efficient methods to increase the proteins production [5, 44]. The original promoter P_*hpaII*_ in pMA5 was a relatively strong promoter. In order to further increase the expression of BcManA, two other strong promote P43 and P_*lapS*_ were selected. Promoter P43 was a well characterized constitutive strong promoter and widely used in expression of many proteins in *B. subtilis* [5, 44]. Whereas, promoter P_*lapS*_ was an artificial hybrid promoter containing P_*luxS*_ and the − 10 region from *B. licheniformis* and the − 35 region of P_*Apr*_ from *B. subtilis*, which was confirmed experimentally to be stronger than P43 for β-Gal secretory expression in *B. subtilis* [75]. The strains *B. subtilis* WB600-8 and *B. subtilis* WB600-9 containing these two strong promoters and SP_*lipA*_ were also cultured in 2 × SR medium by flask shaking at 37 °C for 48 h, and the extracellular activity was assayed (Table 5). The highest enzyme activity of 908 U ml^−1^ was produced by *B. subtilis* WB600-8 (P43) which was higher than that produced by *B. subtilis* WB600-3 (P_*hpaII*_). Similar result was also found in the expression of *B. licheniformis* DSM13 β-mannanase and the activity of 1274 U ml^−1^ and 711 U ml^−1^ was produced by P43 and P_*hpaII*_ after 72 h cultivation, respectively [5]. The activity of 222 U ml^−1^ was determined from *B. subtilis* WB600-9 (P_*lapS*_) which was only about 24.4% of that from *B. subtilis* WB600-8 (P43). However, for the expression of β-Gal, the enzyme production driven by P_*lapS*_ was more than 10 times of that by P43 [75]. This indicated that the strongest promoter P_*lapS*_ might be not suitable for BcManA expression in *B. subtilis* WB600. So it shows that promoter with suitable strength is important for protein expression. Further, we determined the cell growth and BcManA production curve of *B. subtilis* WB600, *B. subtilis* WB600-8 and the original strain *B. clausii* S10 in different medium (Additional file 1: Fig. S2). No mannanase activity was detected in the *B. subtilis* WB600 and *B. clausii* S10 culture supernatant during the entire measuring period. However, small amount of activity was found when *B. clausii* S10 was cultured in the modified Horikoshi-I medium containing locust bean gum, which indicated that the mannanase production in *B. clausii* S10 was inducible. The BcManA yield reached to plateau after 72 h cultivation for both of *B. subtilis* WB600-8 and *B. clausii* S10. And the extracellular activity from *B. subtilis* WB600-8 reached 2374 U ml^−1^ with secretory rate of 98.5% at 72 h, which was much higher than that of native BcManA from *B. clausii* S10 (75 U ml^−1^). Also this enzyme activity was already higher than that produced by other reported *B. subtilis* strains by shake flask (Table 6) [5, 43, 44, 76].

**Table 6:** Secreted production of recombinant β-mannanases in different strains by shake flask

Gene source	Expression hosts	Expression vector	Culture time (h)	Activity (U ml^−1^)	Productivity (U ml^−1^ h^−1^)	Enzyme pH optima	References
*B. clausii* S10	*B. subtilis* WB600	pMA5	72	6041	83.9	9.5	This work
*B. licheniformis* DSM13	*B. subtilis* 1A751	pMA5	72	2207	30.7	5.5	[5]
*B. subtilis* WL-3	*B. subtilis* 168	pJ2788U	15	450	30.0	6.0	[43]
*B. pumilus* Nsic-2	*B. subtilis* 1A751	pBNS2	24	7	0.3	7.0	[44]
*B. subtilis* BCC41051	*B. megaterium*	pXb	24	359	15.0	7.0	[76]
*Bispora* sp. MEY-1	*P. pastoris* GS115	pPIC9	48	64	1.3	1.0–1.5	[59]
*A. fumigatus*	*P. pastoris* GS115	pPICZαC	10	61	6.1	5.2	[26]
*A. niger* BK01	*P. pastoris* X33	pPICZαA	96	669	7.0	4.5	[27]
*B. subtilis* MAFIC-S11	*P. pastoris* X33	pPICZαA	72	1106	15.4	6.0	[42]
*A. sulphureus*	*P. pastoris* X33	pPICZαA	96	96	1.0	2.4	[28]
*B. subtilis* G1	*P. pastoris* GS115	pPICZαA	108	224	2.1	6.5	[80]
*A. niger* CBS 513.88	*P. pastoris* GS115	pHBM905M	192	274	1.4	4.5	[61]
*B. subtilis* BS5	*P. pastoris* GS115	pPIC9	96	892	9.3	6.0	[41]
*Bacillus* sp. N16-5	*P. pastoris* GS115	pPIC9K	144	1114	7.7	9.0	[45]
*Penicillium* sp. C6	*P. pastoris* GS115	pPIC9	120	566	4.7	4.5	[66]
*Bacillus sp.* N16-5	*P. pastoris* GS115	pPICZαA	120	32	0.3	10.0	[40]
*Penicillium oxalicum*	*P. pastoris* GS115	pPICZαA	144	84	0.6	4.0	[30]
*B. circulans* NT 6.7	*E. coli* BL21 (DE3)	pET21d	16	37	2.3	6.0	[38]
*B. licheniformis* DSM13	*E. coli* Top10	pFLAG-CTS	4^a^	13	3.4	6.0–7.0	[37]
*Bacillus* sp. N16-5	*E. coli* BL21 (DE3)	pET22b	28	2736	97.7	9.0	[39]
*A. aculeatus* MRC11624	*A. niger* D15	pGT	192	16,596^b^	35.4	3.8	[77]
*A. fumigatus*	*A. sojae*	pAN52-4	144	352	2.4	4.5	[26]
*A. niger* CBS 513.88	*Y. lipolytica*	pINA1296I	48	255	5.3	4.5	[78]
*B. licheniformis* DSM13	*L. plantarum*	pSIP609	12	42	3.5	6.0	[79]
*T. harzianum* MGQ2	*T. reesei* OM9414	pUC19	168	2460	14.6	6.0	[31]

^a^The time is the induction time

^b^The activity defined as the amount of enzyme required to convert one mole of substrate per second and reported in katal (nkat ml^−1^)

### Optimization for high-level secreted expression of BcManA in *B. subtilis*

**Fig. 6:**
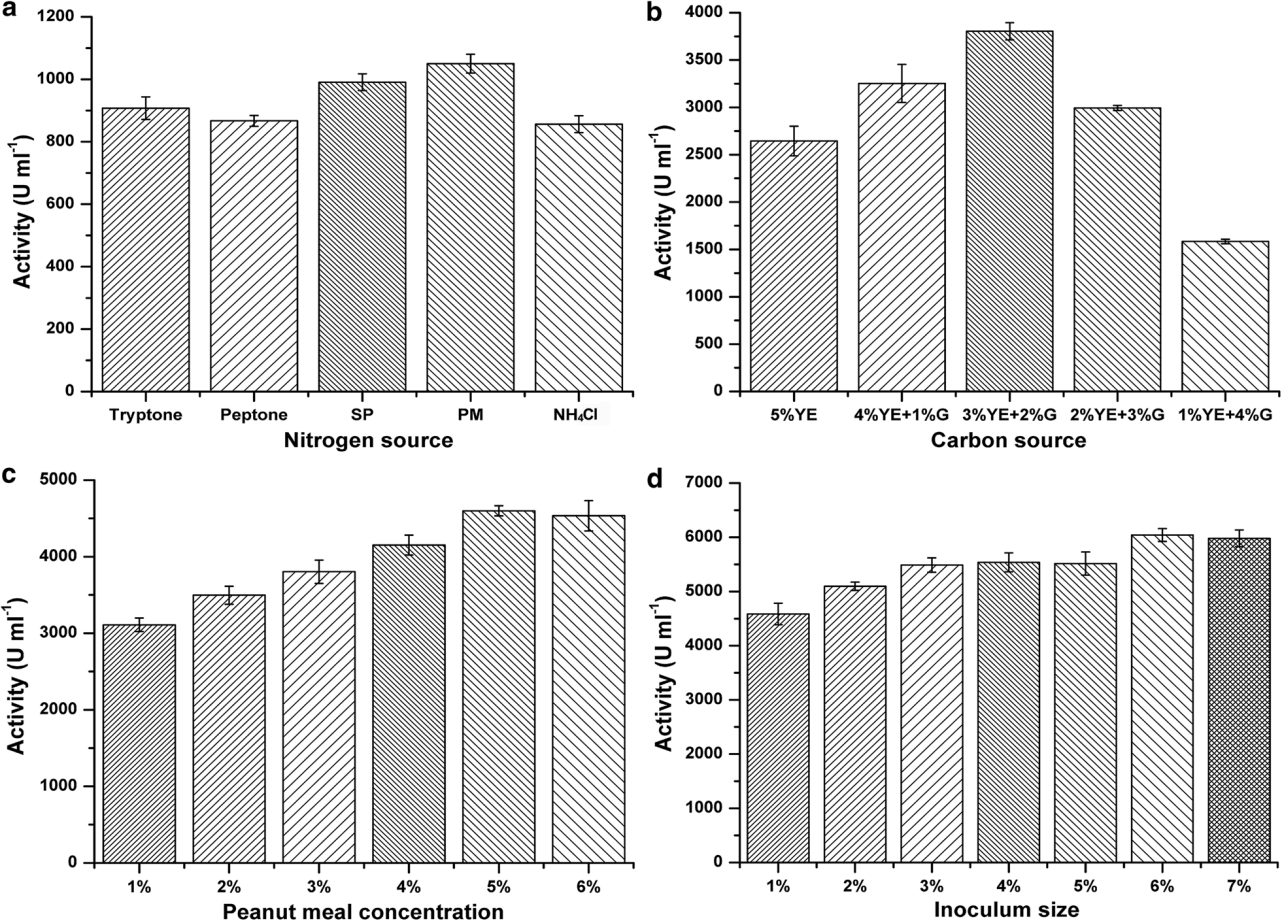
Extracellular β-mannanase activity of the recombinant *B. subtilis* WB600-8 cultured in 50 ml of various modified 2 × SR mediums. **a** Different nitrogen sources with 3% concentration in 2 × SR medium; **b** different proportions substitution of yeast extract (YE) by glucose (G) in the modified 2 × SR medium with 3% peanut meal as nitrogen source; **c** different peanut concentration in the modified 2 × SR medium with 3% yeast extract and 2% glucose; **d** different doses of inoculation in the final modified medium containing 5% peanut meal, 3% yeast extract, 2% glucose and 0.6% K_2_HPO_4_. Error bars represent standard deviations

The medium component was important for industrial enzyme production by microbes. In 2 × SR medium, yeast extract and tryptone which were expensive and generally used only in laboratory were the carbon and nitrogen source. In order to promote the industrial production performance of the recombinant *B. subtilis* WB600-8 for BcManA, different cheap carbon and nitrogen sources were selected for evaluation. Firstly, peptone, soybean powder, peanut meal and the inorganic nitrogen of NH_4_Cl with 3% (w/v) concentration was used to replace tryptone for BcManA expression. As shown in Fig. 6a, obvious extracellular activity was detected after 48 h cultivation and the highest activity (1050 U ml^−1^) was found by using peanut meal which was slightly higher than that by using tryptone (908 U ml^−1^). When the culture time extended to 72 h, a higher activity using peanut meal reached 2645 U ml^−1^ compared with 2374 U ml^−1^ using tryptone. Peanut meals are cheap and abundant by-products of oil extraction from peanut seeds. So they can be used as cheap nitrogen source for β-mannanase production by recombinant *B. subtilis.*

Using peanut meal as nitrogen source, different carbon source such as glucose, glycine, corn starch, soluble starch, konjac flour and dextrin with 2% (w/v) concentration were used instead of yeast extract for BcManA expression. After 48 h cultivation, the highest activity of only 69 U ml^−1^ was determined using glucose as carbon source among these modified mediums, which was at very low level compared with that using yeast extract. These results indicated that yeast extract might be important for BcManA production in *B. subtilis*. On the other hand, we found that the cell concentration in the glucose medium was observably higher than that in the yeast extract medium, along with pH decrease. This phenomenon indicated that glucose in medium could improve *B. subtilis* cells growth to high biomass, however, which resulted in more acid by-products and low level of protein expression. Nevertheless, yeast extract should be partially substituted by cheap carbon source such as glucose to balance the protein expression and cell growth. Different proportions substitution of yeast extract by glucose was performed with the total concentration of 5% (w/v). As shown in Fig. 6b, the highest activity of 3804 U ml^−1^ was found after 72 h cultivation in the medium containing 3% yeast extract and 2% glucose, which was about 2.5 times as much as that only using 5% yeast extract. Further, the effect of peanut meal concentration on BcManA expression was also evaluated. As shown in Fig. 6c, the highest yield of 4599 U ml^−1^ was obtained after 72 h when 5% peanut meal was used in the modified medium. In addition, the inoculation amount is also an important fact for protein expression. The extracellular enzyme yield under different inoculation amount was detected using the optimally modified 2 × SR medium (5% peanut meal, 3% yeast extract, 2% glucose, 0.6% K_2_HPO_4_, pH 7.2). Finally, the highest activity was determined to be 6041 U ml^−1^ after 72 h cultivation when the inoculum size of 6% (v/v) was used (Fig. 6d), which was about 80 times that of native BcManA from *B. clausii* S10.

In the past decade, many strains such as *Bacillus* sp., *P. pastoris*, *E. coli*, *Aspergillus* sp. and some least frequently used strains have been applied for β-mannanases production (Table 6). Among these reports, *B. subtilis* and *P. pastoris* are the most commonly used host strains for β-mannanases expression, and most of the β-mannanases had neutral or acid optimal pH. Song et al. reported the secreted expression of β-mannanase from *B. licheniformis* DSM13 in *B. subtilis* 1A751 through protein synthesis and secretion optimization strategy, and they improved the final production to 2207 U ml^−1^ by shake flask which was considered to be the highest among the reported *Bacillsu* strains [5]. Our result showed a yield of 6041 U ml^−1^ which was about 2.7 times as much as that expressed in *B. subtilis* 1A751, which indicating the highest β-mannanase yield produced by *Bacillus* strains by shake flask. Compared to *Bacillus*, much more secreted expression of β-mannanase was performed in *P. pastoris* strains (Table 6). The highest yield by shake flask was 1114 U ml^−1^ with productivity of only 7.7 U ml^−1^ h^−1^ after 144 h cultivation in *P. pastoris* GS115 using pPIC9K vector [55]. Besides these commonly used strains, some other host strains such as *A. niger* [77], *A. sojae* [26], *Yarrowia lipolytica* [78], *Lactobacillus plantarum* [79] and *Trichoderma reesei* [31] were also used for β-mannanases production. The extracellular activity of β-mannanase from *A. aculeatus* MRC11624 was found to be 16,596 nkat ml^−1^ (about 996 U ml^−1^) after 192 h cultivation in *A. niger* D15 [77]. Higher activity of 2460 U ml^−1^ with productivity of 14.6 U ml^−1^ h^−1^ was obtained for ThMan5 after 72 h cultivation in *T. reesei* QM9414A [31]. In the present study, combined with selection of suitable signal peptide for efficient secretory, strong promoters to enhance the transcription and translation, and optimization of medium for strain growth and protein expression, the extracellular activity of BcManA reached 6041 U ml^−1^ with productivity of 83.9 U ml^−1^ h^−1^ after 72 h cultivation by flask shake, which was higher than those of not only *Bacillus* strains but also all other reported strains by flask shake fermentation. Also the modified 2 × SR medium was cheaper than the normally used 2 × SR medium and could be applied for other protein expression in *B. subtilis*. Moreover, a much higher production of BcManA would be obtained by cultivating this strain in fed-batch fermentors with process optimization.

## Conclusions

The thermo-alkaline β-mannanase (BcManA) gene from *B. clausii* was cloned and heterologous expressed. The purified recombinant BcManA showed high catalytic activity on konjac glucomannan and locust bean gum under alkaline and high temperature conditions. Overall consideration of pH and temperature performance, BcManA distinguished from other reported β-mannanases and was a promising thermo-alkaline β-mannanase for potential industrial application. Combined with medium optimization and selection of suitable signal peptide and strong promoters, the extracellular BcManA yield of 6041 U ml^−1^ was obtained in *B. subtilis* WB600 with constitutive expression vector, which representing the highest yield reported to date by flask shake. This would significantly cut down the production cost of this enzyme and provide a guideline for secretory expression of other β-mannanases in *B. subtilis*.

## Additional files

**Additional file 1: Table S1.** Primers for expression plasmid construction with different signal peptides by modified Gibson method. **Table S2.** Primers for expression plasmid construction with different promoters by modified Gibson method.

**Additional file 2: Fig. S1.** The screening result of the positive mannanase clone on the LB agar plate containing locust bean gum. The colony pointed out by white arrow showed transparent zone which indicated positive mannanase activity. **Fig. S2.** Time profiles for mannanase activity by *B. clausii* S10, *B. subtilis* WB600 and the recombinant *B. subtilis* WB600-8. *Black square*–extracellular activity by *B. subtilis* WB600-8 in 2 × SR medium; *black circle*–extracellular activity by *B. clausii* S10 in modified Horikoshi-I medium containing konjac glucomannan; *white square*–the OD_600_ value of *B. subtilis* WB600-8; *white circle*–the OD_600_ value of *B. clausii* S10 in modified Horikoshi-I medium containing konjac glucomannan; *white triangle*–the OD_600_ value of *B. clausii* S10 in Horikoshi-I medium; *white inverted triangle*–the OD_600_ value of *B. subtilis* WB600 in 2 × SR medium. Due to no activity was detected in the entire measuring period, the enzyme production curves of *B. subtilis* WB600 and *B. clausii* S10 in Horikoshi-I medium were not shown on this figure.
