# Editorial expression of concern:Characterization and high-efciency secreted expression in bacillus subtilis of a thermo-alkaline β-mannanase from an alkaliphilic bacillus clausii strain S10

**DOI:** 10.1186/s12934-024-02580-1

**Published:** 2024-11-27

**Authors:** Cheng Zhou, Yanfen Xue, Yanhe Ma

**Affiliations:** 1 State Key Laboratory of Microbial Resources,Institute of Microbiology, Chinese Academy of Sciences, Beijing, 100101 China; 2 National Engineering Laboratory for Industrial Enzymes, Institute of Microbiology,Chinese Academy of Sciences, Beijing, 100101 China

Editorial Expression of Concern: Microb Cell Fact (2018) 17:124.10.1186/s12934-018-0973-0

The Editor-in-Chief is issuing an editorial Expression of Concern for this article. Concerns have been raised that the sequencing information for BcManA is not included in the article and has not been deposited in a publicly available database. Readers are therefore advised to interpret the results and conclusions with caution. The authors have not responded to correspondence from the Publisher about this statement.

