# Supplementary material for: Characterization and high-efficiency secreted expression in Bacillus subtilis of a thermo-alkaline β-mannanase from an alkaliphilic Bacillus clausii strain S10
Source: Microb Cell Fact. 2018 Aug 11;17:124. doi: 10.1186/s12934-018-0973-0 (PMC6087540; doi:10.1186/s12934-018-0973-0)
Supplement: Supplementary file 1 — Additional file 1: Table S1. Primers for expression plasmid construction with different signal peptides by modified Gibson method. Table S2. Primers for expression plasmid construction with different promoters by modified Gibson method. [file 12934_2018_973_MOESM1_ESM.docx]

**Additional file 1: Table S1.** Primers for expression plasmid construction with different signal peptides by modified Gibson method.

| **Primer name** | **Primer sequences (5’**–**3’)** |
| --- | --- |
| SP*_ori_*-F | GTTGGAGCACTAGTGGTGGTGTTGGTTTTGTTTGTATACAGTAGCGGTTTAGCATCTGCACAAAGCGGCTTTCACGTAAAAGGTACAGAGTTG |
| SP*_ori_*-R | TATACAAACAAAACCAACACCACCACTAGTGCTCCAACCCATTGTAACCGCTTACGATTCATagctagcttgagctcgactctagaggATCCCC |
| SP*_lipA_*-F | TGTAACAATTTTGATGCTGTCTGTTACATCGCTGTTTGCGTTGCAGCCGTCAGCAAAAGCCCAAAGCGGCTTTCACGTAAAAGGTACAGAGTTG  CGATGTAACAGACAGCATCAAAATTGTTACAAGTGCAATGATCCTTCTTTTTACAAATTTCATagctagcttgagctcgactctagaggatcccC |
| SP*_lipA_*-R |  |
| SP*_lipB_*-F | TTGTTTATCGCTGATTCTATCTGTTTTAGCCGCTCCGCCGTCTGGCGCAAAAGCTCAAAGCGGCTTTCACGTAAAAGGTACAGAG  AAACAGATAGAATCAGCGATAAACAAATAATGAATGCCATAAGTACTTTTTTCATagctagcttgagctcgactctagaggatccc |
| SP*_lipB_*-R |  |
| SP*_aprE_*-F | TGTTGTTTGCGTTAACGTTAATCTTTACGATGGCGTTCAGCAACATGTCTGCGCAGGCTCAAAGCGGCTTTCACGTAAAAGGTACAGAG  TCGTAAAGATTAACGTTAACGCAAACAACAAGCTGATCCACAATTTTTTGCTTCTCATagctagcttgagctcgactctagaggatccc |
| SP*_aprE_*-R |  |
| SP*_amyL_*-F | GATTGCTGCCGCTGTTATTTGCGCTCATCTTCTTGCTGCCTCACTCTGCAGCAGCGGCGCAAAGCGGCTTTCACGTAAAAGGTACAGAG  AGATGAGCGCAAATAACAGCGGCAGCAATCGGGCATAAAGCCGTTTGTGTTGTTTCATagctagcttgagctcgactctagaGGATCCC |
| SP*_amyL_*-R |  |
| SP*_amyE_*-F | CGTTATTCGCTGGATTTTTATTGCTGTTTCATTTGGTTCTGGCAGGACCGGCGGCTGCGAGTGCTCAAAGCGGCTTTCACGTAAAAGGTACAGAGTT |
| SP*_amyE_*-R | GAAACAGCAATAAAAATCCAGCGAATAACGGCAGTAAAGAGGTTTTGAATCGTTTTGCAAACATagctagcttgagctcgactctagaggatcccc |

**Additional file 1: Table S2.** Primers for expression plasmid construction with different promoters by modified Gibson method.

| **Primer name** | **Primer sequence (5’–3’)** |
| --- | --- |
| P43-F1 | TATAGTGTGTTATACGAGCTCAGCATTATTGAGTGGATGAT |
| P43-R1 | AACTCATATGTAAATATATATTCCTCCTTTGGTACCGCTATC |
| P43-F2 | AAAGGAGGAATATATATTTACATATGAGTTATGCAGTTTGTAG |
| P43-R2 | ATAATGCTGAGCTCGTATAACACACTATACTTTATATTCATA |
| P*_lapS_*-F1 | ATAGTGTGTTATACGAGCTCAGGCCTTAACTCACATTAATTG |
| P*_lapS_*-R1 | ACTCATATGTAAATCGTTCATGTCTCCTTTTTTATGTACTGTG |
| P*_lapS_*-F2 | AAGGAGACATGAACGATTTACATATGAGTTATGCAGTTTGTAG |
| P*_lapS_*-R2 | GTTAAATGCTCCTGAGTATAACACACTATACTTTATATTCATA |
