# Supplementary material for: Characterization and high-efficiency secreted expression in Bacillus subtilis of a thermo-alkaline β-mannanase from an alkaliphilic Bacillus clausii strain S10
Source: Microb Cell Fact. 2018 Aug 11;17:124. doi: 10.1186/s12934-018-0973-0 (PMC6087540; doi:10.1186/s12934-018-0973-0)
Supplement: Supplementary file 2 — Additional file 2: Fig. S1. The screening result of the positive mannanase clone on the LB agar plate containing locust bean gum. The colony pointed out by white arrow showed transparent zone which indicated positive mannanase activity. Fig. S2. Time profiles for mannanase activity by B. clausii S10, B. subtilis WB600 and the recombinant B. subtilis WB600-8. Black square–extracellular activity by B. subtilis WB600-8 in 2 × SR medium; black circle–extracellular activity by B. clausii S10 in modified Horikoshi-I medium containing konjac glucomannan; white square–the OD600 value of B. subtilis WB600-8; white circle–the OD600 value of B. clausii S10 in modified Horikoshi-I medium containing konjac glucomannan; white triangle–the OD600 value of B. clausii S10 in Horikoshi-I medium; white inverted triangle–the OD600 value of B. subtilis WB600 in 2 × SR medium. Due to no activity was detected in the entire measuring period, the enzyme production curves of B. subtilis WB600 and B. clausii S10 in Horikoshi-I medium were not shown on this figure. [file 12934_2018_973_MOESM2_ESM.docx]

**Additional figures:**


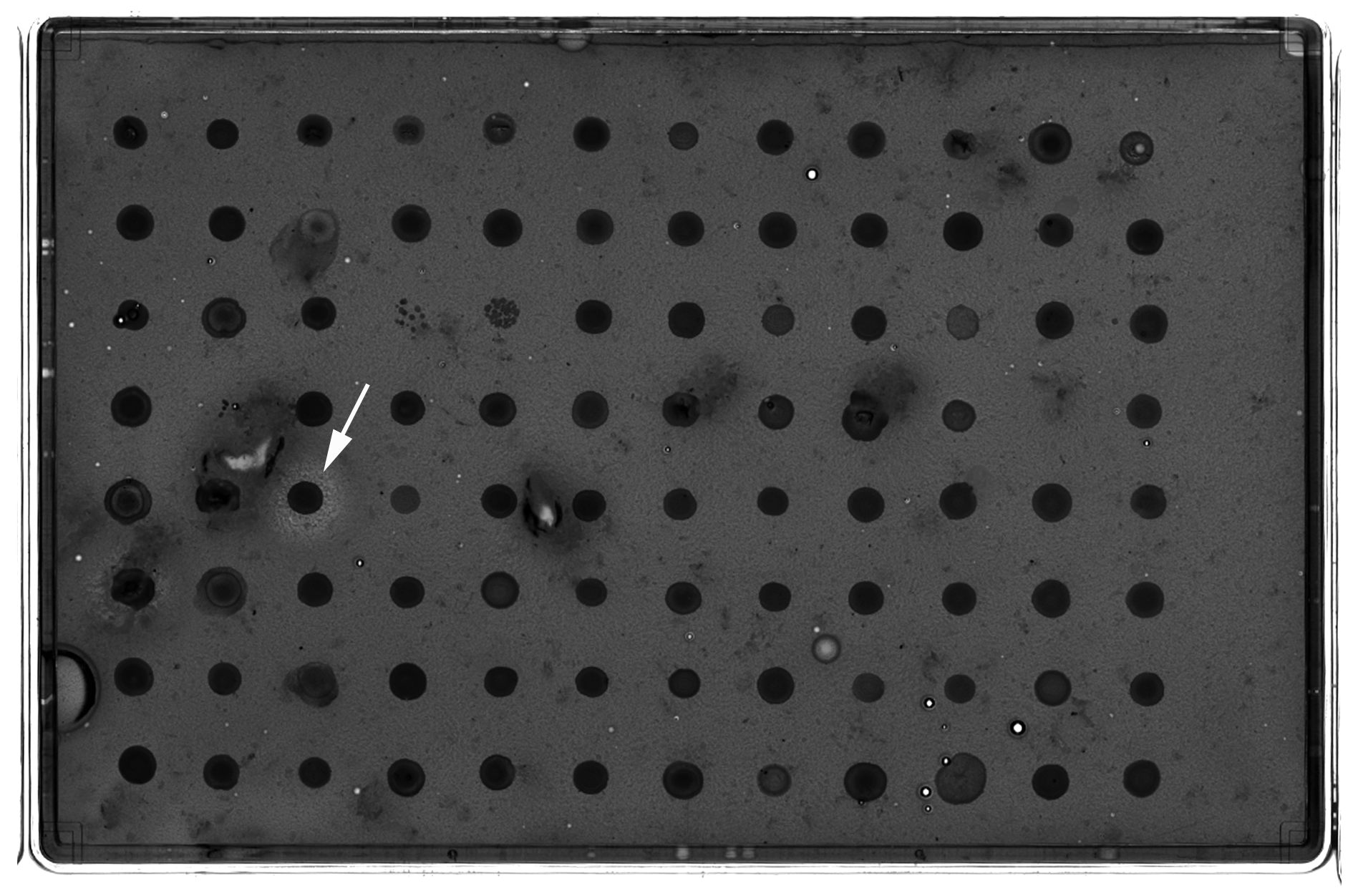


**Additional file 2: Fig. S1.** The screening result of the positive mannanase clone on the LB agar plate containing locust bean gum. The colony pointed out by white arrow showed transparent zone which indicated positive mannanase activity.





**Additional file 2: Fig. S2.** Time profiles for mannanase activity by *B. clausii* S10, *B. subitilis* WB600 and the recombinant *B. subtilis* WB600-8. *Black square*–extracellular activity by *B. subtilis* WB600-8 in 2×SR medium; *black circle*–extracellular activity by *B. clausii* S10 in modified Horikoshi-I medium containing konjac glucomannan; *white square*–the OD_600_ value of *B. subtilis* WB600-8; *white circle*–the OD_600_ value of *B. clausii* S10 in modified Horikoshi-I medium containing konjac glucomannan; *white triangle*–the OD_600_ value of *B. clausii* S10 in Horikoshi-I medium; *white inverted triangle*–the OD_600_ value of *B. subtilis* WB600 in 2×SR medium. Due to no activity was detected in the entire measuring period, the enzyme production curves of *B. subtilis* WB600 and *B. clausii* S10 in Horikoshi-I medium were not shown on this figure.
